# Virulence Factors and in-Host Selection on Phenotypes in Infectious Probiotic Yeast Isolates (*Saccharomyces ‘boulardii’*)

**DOI:** 10.3390/jof7090746

**Published:** 2021-09-11

**Authors:** Alexandra Imre, Renátó Kovács, Kitti Pázmándi, Dániel Nemes, Ágnes Jakab, Tünde Fekete, Hanna Viktória Rácz, Ilona Dóczi, Ildikó Bácskay, Attila Gácser, Károly Kovács, László Majoros, Zoltán Farkas, István Pócsi, Walter P. Pfliegler

**Affiliations:** 1 Department of Molecular Biotechnology and Microbiology, University of Debrecen, Egyetem tér 1., H4032 Debrecen, Hungary; imre.alexandra@science.unideb.hu (A.I.); jakab.agnes@science.unideb.hu (Á.J.); racz.hannaviktoria@science.unideb.hu (H.V.R.); pocsi.istvan@science.unideb.hu (I.P.); 2 Kálmán Laki Doctoral School of Biomedical and Clinical Sciences, University of Debrecen, Egyetem tér 1., H4032 Debrecen, Hungary; 3 Department of Medical Microbiology, University of Debrecen, Egyetem tér 1., H4032 Debrecen, Hungary; kovacs.renato@med.unideb.hu (R.K.); major@med.unideb.hu (L.M.); 4 Faculty of Pharmacy, University of Debrecen, Egyetem tér 1., H4032 Debrecen, Hungary; 5 Department of Immunology, University of Debrecen, Egyetem tér 1., H4032 Debrecen, Hungary; pazmandi.kitti@med.unideb.hu (K.P.); fekete.tunde@med.unideb.hu (T.F.); 6 Department of Pharmaceutical Technology, University of Debrecen, Egyetem tér 1., H4032 Debrecen, Hungary; nemes.daniel@pharm.unideb.hu (D.N.); bacskay.ildiko@pharm.unideb.hu (I.B.); 7 Doctoral School of Nutrition and Food Sciences, University of Debrecen, Egyetem tér 1., H4032 Debrecen, Hungary; 8 Institute of Clinical Microbiology, University of Szeged, Semmelweis Str. 6, H6725 Szeged, Hungary; doczi.ilona@med.u-szeged.hu; 9 HCEMM-USZ Fungal Pathogens Research Group, Department of Microbiology, Faculty of Science and Informatics, University of Szeged, Közép Fasor 52, H6725 Szeged, Hungary; gacsera@bio.u-szeged.hu; 10 MTA-SZTE “Lendület” Mycobiome Research Group, University of Szeged, Közép Fasor 52, H6725 Szeged, Hungary; 11 Synthetic and Systems Biology Unit, Institute of Biochemistry, Biological Research Centre, Temesvári Krt. 62, H6726 Szeged, Hungary; kovacs.karoly@brc.hu (K.K.); farkas.zoltan@brc.hu (Z.F.); 12 HCEMM-BRC Metabolic Systems Biology Lab, Biological Research Centre, Temesvári Krt. 62, H6723 Szeged, Hungary

**Keywords:** *Saccharomyces boulardii*, opportunistic pathogen, microevolution, antimycotic, virulence

## Abstract

*Saccharomyces* yeast probiotics (*S. ‘boulardii’*) have long been applied in the treatment of several gastrointestinal conditions. Despite their widespread use, they are rare opportunistic pathogens responsible for a high proportion of *Saccharomyces* mycosis cases. The potential virulence attributes of *S. ‘boulardii’* as well as its interactions with the human immune system have been studied, however, no information is available on how these yeasts may change due to in-host evolution. To fill this gap, we compared the general phenotypic characteristics, cell morphology, virulence factors, epithelial and immunological interactions, and pathogenicity of four probiotic product samples, two mycosis, and eight non-mycosis samples of *S. ‘boulardii’*. We assessed the characteristics related to major steps of yeast infections. Mycosis and non-mycosis isolates both displayed novel characters when compared to the product isolates, but in the case of most virulence factors and in pathogenicity, differences were negligible or, surprisingly, the yeasts from products showed elevated levels. No isolates inflicted considerable damage to the epithelial model or bore the hallmarks of immune evasion. Our results show that strains in probiotic products possess characteristics that enable them to act as pathogens upon permissive conditions, and their entry into the bloodstream is not due to active mechanisms but depends on the host. Survival in the host is dependent on yeast phenotypic characteristics which may change in many ways once they start evolving in the host. These facts call attention to the shortcomings of virulence phenotyping in yeast research, and the need for a more thorough assessment of probiotic use.

## 1. Introduction

Throughout history, *Saccharomyces cerevisiae* has been an indispensable fungus in agriculture and the food industry [1,2]. Besides the widespread and diverse applications of this yeast in various industries, a subtype of the species, namely *S. ‘boulardii’* is applied commonly as a probiotic both for human use and even for livestock [3,4,5,6,7]. It is by far the leading probiotic yeast in the world [8] and its probiotic properties have been demonstrated in more than 80 randomized clinical trials for the strain *S. ‘boulardii’* CNCM I-745 [6]. Nowadays this probiotic yeast is used for the treatment of *Clostridium difficile*-related and antibiotic-associated diarrhea [7], and to improve the symptoms of irritable bowel syndrome (IBS) [9,10]. Previous studies analyzing the whole genome sequences of *S. ‘boulardii’* have found that isolates from different sources show little variation and most of them have a diploid euploid genome [11,12].

Surprisingly, this widely used and thoroughly researched probiotic yeast is also an opportunistic pathogen. Among all non-*Candida* yeasts, the genus *Saccharomyces* causes the highest number of infections and in fact, the *S. ‘boulardii’* probiotic yeast is responsible for most of these [13]. Furthermore, this yeast is the most commonly pathogenic probiotic microorganism even when bacterial species are also considered [13]. Infections with the probiotic yeast, as in the case of all probiotics, occur mainly among immunocompromised or severely ill/hospitalized patients, infants, and elderly people. In recent years a growing number of such cases were reported in clinical case reports e.g., [14,15,16,17]. Currently no precise data are available on the frequency of these infections, largely due to the earlier lack of clinically applicable subtyping methods that can reliably connect a yeast infection to the *S. ‘boulardii’* probiotic products itself. Thus, we optimized a multiplex PCR protocol to differentiate clinical *S. ‘boulardii’* isolates from other yeasts [16] utilizing the presence of characteristic microsatellite and retrotransposon polymorphisms in *S. ‘boulardii’* that distinguishes it from other *S. cerevisiae* [11,18,19,20]. We demonstrated that probiotic-derived clinical yeasts are common among *S. cerevisiae* isolates collected from a single Hungarian hospital over the course of three years (seven isolates, including two fungemia cases, were probiotic-derived out of 15 *S. cerevisiae* infections [16]).

Based on the above-mentioned high proportion of *S. ‘boulardii’* both among *Saccharomyces* mycosis cases and among probiotic infections (half of all described probiotic infections up until 2018) [13], earlier studies have aimed to identify the pathomechanism and the important virulence factors of the probiotic yeasts using various methods, often comparing them to a limited number of other *Saccharomyces* isolates. In their review on pathogenic *Saccharomyces*, Anoop et al. [21] stated that the methodical approach used for assessing *S. cerevisiae* strains’ pathogenic potential should combine genetic in vitro and in vivo analyses, defining comparison of results between virulent and non-virulent strains. The authors suggest high thermotolerance [22,23,24,25], pseudo hyphal [23,24,25], and invasive growth [23,25,26], as well as enzymatic expression (protease and phospholipase activity) [24,25], adhesion to mammalian cells [23,27,28,29], activation of innate immunity (cytokine production, phagocytosis) [28,29] and modulation of oxidative stress response [30] as features to be tested.

Among the previous virulence studies to include probiotic yeasts, McCullogh et al. [31] found an ‘intermediate virulence’ of probiotic yeast strains in mouse model, similar to Yáñez et al. [28]. Both studies compared *S. ‘boulardii’* to other, not closely related, partially clinical isolates. Klingberg et al. [23] found no specific virulence factors (after testing pseudohyphal growth, invasivity, adhesion and damage to the epithelium) that differentiated clinical *Saccharomyces* from others, including three commercial *S. ‘boulardii’* strains that all showed no invasivity and pseudohyphal growth only on low-nitrogen medium. de Llanos et al. [32] used various mouse models, testing, among others, a single *S. ‘boulardii’* commercial isolate. Their work underlined a higher virulence in the case of baker’s yeasts and *S. ‘boulardii’*, but not in other yeasts, when immunosuppressed mice were experimentally infected. In general, the probiotic yeast showed intermediate virulence. In a later study, a single probiotic strain, along with unrelated clinical ones, was found to be unable to cross the epithelial barrier in vitro, using the Caco-2 epithelial model [27]. Llopis et al. [33] compared a single isolate from an *S. ‘boulardii’* probiotic with other, non-related probiotic and food supplement *Saccharomyces* yeasts with two virulent non-*boulardii* clinical isolates. They stated that the probiotic *S. ‘boulardii’* strain was capable of growth at 42 °C and exhibited moderate virulence factors in some of the assays (e.g., pseudohyphal growth, phospholipase secretion) when compared with other yeast samples.

Despite the many clinical case reports, literature reviews, systematic reviews, or primary research papers on the probiotic yeasts’ phenotype compared with other *S. cerevisiae*, comparisons of actual *S. ‘boulardii’* yeast isolates from patients and from products are extremely scarce and inconclusive. To our knowledge, only two such studies have been published. Pfliegler et al. [34] compared the phenotypic characteristics of a single probiotic-derived clinical isolate from Hungary with a batch of a locally available product and found highly similar characteristics in these, in contrast to the more conspicuous in-host changes manifesting in baker’s yeast derived clinical isolates. Additionally, Peter et al. [12] listed 24 isolates (including commercial and clinical isolates) in the *S. ‘boulardii’* subclade and recorded growth under various conditions for altogether 1011 *S. cerevisiae* isolates. However, they did not discuss phenotypic differences of commercial/clinical counterparts within clades. It thus remains unknown whether the phenotypic characteristics and virulence attributes of the probiotic yeast commercial isolates are representative of the whole clade. In other words, we do not know whether *S. ‘boulardii’* can evolve novel characteristics under selection in the host environment, either during benign colonization or in the cases of infection. The *S. ‘boulardii’* subclade contains very closely related isolates, but it is not entirely genetically uniform [12], and it was also shown that probiotic products, among other commercial yeasts, may contain phenotypically diverse subclonal lineages [35]. This standing genetic and phenotypic variation in the probiotic yeast may plausibly lead to in-host selection and evolution (compare with [34]) that needs to be studied.

Our aim in this study was to use our collection of probiotic yeasts and probiotic-derived clinical yeast isolates (both pathogenic and likely commensal ones) to compare their phenotypes, virulence factors, immunogenicity, and pathogenicity in an invertebrate model, to identify traits that are selected for in *S. ‘boulardii’* during survival and colonization of the human host. To this end we assessed the traits of four subclone cultures of probiotics from two manufacturers along with ten probiotic-derived clinical isolates, all collected in Hungary.

## 2. Materials and Methods

### 2.1. Isolates and Patient Data

Isolated subclone lines were established from two batches of two different, locally available *S. ‘boulardii’* products, and ten clinical isolates from the university clinics of Debrecen and Szeged (Hungary). For the latter, detailed patient and isolation data were available. Patient data were handled in accordance with EU, state, and local regulations with a clinical study ethics approval from the Regional and Institutional Research Ethics Council of Debrecen (DE RKEB/IKEB 5194-2019). Stocks of the clinical and commercial isolates were saved at −70 °C in YPD broth (VWR Chemicals, Solon, OH, USA, pH 5.8) supplemented with 30% glycerol. These commercial and clinical isolates were subcultured only once (multiple single-cell bottlenecks were avoided) to prevent accumulation of potential geno- and phenotypic changes in the samples due to the phenomenon of clonal heterogeneity that was recently demonstrated for yeast strains [35]. A list of the isolates used in this study is presented in Table 1.

### 2.2. Genotyping

Genomic DNA was isolated according to Hanna and Xiao [36] and stored standardized to 100 ng/µL in 1 × TE buffer at −20 °C. The multiplex PCR method developed for the identification of probiotic derived yeast infections was used to compare previously described infectious *S. ‘boulardii’*, the three novel samples from the University Clinic of Szeged, and the four probiotic product isolates used here. Conditions of the multiplex PCR, gel electrophoresis, and comparison of band patterns followed the published protocol [16] and the GoTaq G2 (Promega, Madison, WI, USA) polymerase enzyme was used. Briefly, the method combines interdelta, microsatellite (*YLR177w*, *YOR267c*), and as a control, ITS 1–4 primer pairs in a single PCR reaction, yielding products of characteristic length for the probiotic yeasts. We further subjected the three novel clinical samples to multi-locus sequence typing (MLST) to corroborate multiplex results. We sequenced four nuclear genes (*CCA1*, *CYT1*, *HMX1*, and *NUP116*), and the nuclear internal transcribed spacer (ITS) region using Pwo polymerase (Sigma-Aldrich, St. Louis, MO, USA) with the primers and conditions described in [16]. PCR products were cleaned with the Illustra GFX PCR DNA and Gel Band Purification Kit (GE Healthcare, Chicago, IL, USA) and sequenced bidirectionally using the primers used for amplification (Microsynth AG, Balgach, Switzerland). Amplified and sequenced products spanned the whole *CYT1* and *HMX1* gene, other genes were partial. Reads were checked and edited using Chromas 2.6.5. (Technelysium, Brisbane, Australia). Sequences were deposited in GenBank (MZ712202–MZ712213). For comparison with sequences in our previous work [16], sequences were aligned (using MUSCLE) and alignments were analyzed using MEGA X [37]. Alignments are deposited in FigShare (doi:10.6084/m9.figshare.15145020).

### 2.3. Phenotyping

*Colony morphology and invasivity*. Colony phenotypes were observed after plating 10 µL of overnight cultures (on YPD, 25 °C) set to OD_600_ = 0.1 onto YPD (VWR, 2% glucose, 2% peptone, 1% yeast extract), SD (synthetic dextrose, 2% glucose, 0.17% yeast nitrogen base without amino acids, without ammonium sulphate (Alpha Aesar—ThermoFischer, Kandel, Germany), 0.5% ammonium sulfate), SLAD (synthetic low-ammonium (50 μM) dextrose medium, as SD except for ammonium sulfate concentration), and SLG (synthetic low-glucose (0.1%) medium, as SD except for glucose concentration) agar plates (2% agar in all agar media in this study). Vented plastic plates with 90 mm diameter and 14 mm height were used (VWR). Plates were incubated for 10 days at 37 °C (with agar surface facing down) after which plates were visually scored for colony phenotypes. Subsequently, colonies were washed under tap water, observed with transillumination (visual light) to score invasivity into the agar media. Colony senescence was observed by inoculating the samples as above onto GlyYP (glycerol 2%, yeast extract 1%, peptone 2%, agar 2%) + 0.1% glucose plates, and visually observing the morphology of colonies after incubation at 37 °C for 30 days.

*Killer activity/sensitivity*. Killer toxin production and sensitivity were evaluated using a K1 and a K2 toxin producer (NCYC 232; NCYC 738, respectively) as well as a sensitive wine yeast strain (NCYC 1006) [38] as control. Killer activity tests were performed by dropping 20 μL OD_600_ = 0.1 suspensions of the killer controls and of the *S. ‘boulardii’* isolates pre-cultured as above onto the surface of YPD agar plates supplemented with 0.003 g/L methylene blue, buffered to pH 4.5 with citrate-phosphate buffer. Plates were incubated for 2 days at 25 °C, then suspensions of the pre-cultured sensitive test strain (1.0 McFarland, 500 µL) were sprayed on the plates, followed by 2 days incubation at 25 °C. Killer sensitivity was tested by making such lawns of the *S. ‘boulardii’* around killer 1 and killer 2 strain colonies. Killer positive activity was registered when a halo of growth inhibition was produced, including the presence of a dark blue zone of dead cells around the edge of the inhibition zone.

*Petite frequency*. The frequency of petite mitochondrial mutants in various *S. ‘boulardii’* was assessed by plating overnight cultures (on YPD, 25 °C) onto GlyYP + 0.1% glucose agar plates with cell densities of approx. 100/plate (after cell counting in a Thoma hemocytometer). Vented plastic plates with 90 mm diameter and 14 mm height were used. Plates were incubated for 10 days at 30 °C (with agar surface facing down) for potential petite mutants on GlyYP. Presumed petites were transferred to YPD and after overnight culturing, were inoculated onto GlyYP plates without glucose. The subclone samples unable to grow on glucose-free agar were scored as petites.

*Sporulation and MAT locus typing*. Sporulation capability was tested by inoculating overnight YPD agar cultures onto potassium acetate sporulation medium (0.05% glucose, 1% potassium acetate, 0.1% yeast extract, 2% agar). Sporulation was evaluated after 5 days of incubation at 25 °C and at 37 °C, using phase contrast microscopy and 400× magnification. The mating type locus (MAT) on chr. III was amplified using the primers (5′-AGTCACATCAAGATCGTTTATG-3′), (5′-GCACGGAATATGGGACTACTTCG-3′), and (5′-ACTCCACTTCAAGTAAGAGTTTG-3′) [39]. For each reaction, 0.25 unit GoTaq G2 polymerase, 20 ng of genomic DNA, 10 pmols of each primer, and 0.2 mM each dNTP in 12.5 μL end volume were used. Reactions were carried out in a C1000 Touch thermocycler. Gel electrophoresis was conducted in 2% low electroendosmosis TBE agarose gel stained with GelRed (Fremont, CA, USA), at 110 V for 30 min followed by visual inspection of gel photographs taken using a UV transilluminator. Bands corresponding to either mating types were identified and used to infer the mating type heterozygosity of the yeasts.

*Growth at high temperature*. Growth at 37–42 °C was evaluated according to [13] using spot plate inoculation. Cells grown overnight at 30 °C in YPD were set to 0.5 McFarland units in water and plated onto YPD in 5 µL drops in a 10× dilution series of approx. 50,000, 5000, 500, 50, and 5 cells. Plates were incubated at various temperatures for 3 days. Growth was scored after visual inspection and comparison with growth at 37 °C (−: no growth, +−: very weak, +: weak, ++: normal, +++ strong growth).

*Susceptibility to antifungal agents*. Minimal inhibitory concentration (MIC) values were determined for three antifungal drugs, fluconazole (Molecula Limited, Newcastle Upon Tyne, UK), amphotericin B (Duchefa Biochemie, Haarlem, The Netherlands), and caspofungin (Molcan, Toronto, Canada). Isolates were grown in pH 7.0, bicarbonate-free RPMI 1640 (Roswell Park Memorial Institute medium, Sigma-Aldrich) and the serial dilution method was used for the experiments in 96 well microtiter plates. Cell concentrations, the use of reference strains, and determination of MIC values after 24 h followed the reference method for broth microdilution antifungal susceptibility testing of yeasts [40].

*Adhesion to plastic surfaces*. To test adhesion to plastic surfaces, yeasts were pre-cultured on YPD overnight, and from suspensions of measured cell densities, ~10,000 cells/mL were inoculated to 3 mL YPD broth into flat bottom polystyrene 12 well plates pre-treated for cell cultures (TPP, Trasadingen, Switzerland). Incubation was carried out for 5 days at 37 °C without shaking, after which cells were washed off 3 times by submerging plates into tap water. Remaining cells were stained with 0.1% crystal violet solution in ddH_2_O (300 µL) for 15 min at room temperature, after which crystal violet solution was removed, cells were washed twice with tap water. The bottoms of the wells were broken from the plates to be viewed under an Olympus BD40 microscope with 20–40× magnification with transmitted light, with representative photographs taken. Morphology was recorded. For each sample, three viewfields were observed on each of the triplicate wells, area of viewfield was calculated, and number of adherent cell clusters for 1 mm^2^ was subsequently calculated based on each individual viewfield. Raw data of measurements were uploaded to FigShare (doi:10.6084/m9.figshare.15109488).

*Extracellular protease activity, phospholipase activity, hemolysis*. Strains were cultured on yeast peptone dextrose medium (YPD; 1% yeast extract, 2% mycological peptone, 2% glucose, 2% agar, pH 5.6) for 2 days. The strains were pre-cultured in liquid YPD medium overnight at 30 °C, then were washed three times with phosphate buffered saline (PBS). Strains were inoculated in 5 μL (5 × 10^4^ cells; protease secretion and hemolysis) and 5 µL (2.5 × 10^6^ cells; phospholipase secretion) of cell suspension in three replicates. We followed hemolytic activity [41] on blood Sabouraud Dextrose Agar (SDA) (4% glucose, 1% pepton, 7.5% sheep blood, 1.5% agar) at 37 °C in 5% CO_2_ for 3 days in Petri dishes with good ventilation to avoid microbial alcohol mediated hemolysis [42]. Hemolytic indices were determined in triplicates after 1 and 2 (α-hemolysis) and 2 and 3 (β-hemolysis) days of incubation. Protease secretion was followed on bovine serum albumin medium (BSA; 0.02% MgSO_4_ · 7 H_2_O, 0.25% K_2_HPO_4_, 0.5% NaCl, 0.1% yeast extract, 2% glucose, 0.25% bovine serum albumin (Sigma-Aldrich), 2% agar) at 37 °C for 2 days (measured after 2 days as well). Phospholipase secretion was investigated on egg yolk medium (EY; 4% glucose, 1% peptone, 1.5% agar, 5.85% NaCl, 0.0555% CaCl_2_, 10% *v*/*v* egg yolk emulsion, the latter containing 2 volumes of physiological saline and 1 volume of egg yolk) at 37 °C for 2 days [25]. Raw data of measurements were uploaded to FigShare (doi:10.6084/m9.figshare.15145020).

*Cell morphology measurements with high-throughput microscopy*. To quantitatively measure the morphology of individual cells of the tested isolates, we applied a previously established protocol [43,44] with modifications. Yeast strains were inoculated into standard 96 well plates (Greiner Bio-One, Kremsmünster, Austria) in YPD at 30 °C in four biological replicates into random positions of separate plates to avoid systematic plate effects. Each plate contained five control wells (BY4743 diploid laboratory strain) in random positions. After reaching the saturation in cell density, each culture was diluted into 500 μL fresh YPD medium in a 96 deep well plate including 0.5 mm glass beads in each well and grown until mid-exponential phase. After that, the cells were fixed in phosphate buffer (pH 6.5) containing 3.7% formaldehyde (Sigma-Aldrich). Fixed cells were washed with PBS and P buffer (10 mM Na_2_HPO_4_ · 12 H_2_O, pH 7.2, 150 mM NaCl). Subsequently we performed fluorescent immunostaining of the cell wall and the nucleus. Actin-staining was omitted due to low reproducibility [45] (Farkas et al., submitted). Staining of the cell wall was performed with 1 mg/mL Alexa-488-conA (Thermo Fisher) solution for 2 h at 4 °C. Nuclei were stained with 350 ng/mL DAPI (Thermo Fisher) in PBS buffer supplemented with 0.1% Triton X-100 (Molar Chemicals, Halásztelek, Hungary) for 30 min at room temperature. The stained cells were diluted and transferred into black clear bottom 96 well plates coated with 1 mg/mL concanavalin A solution (Santa-Cruz Biotechnology, Dallas, TX, USA) and sedimented by centrifugation (1500 rpm for 4 min). Microscopy screening was performed by an Operetta High-Content Imaging System (PerkinElmer Inc., Waltham, MA, USA) using a 63× high numerical aperture objective (Farkas et al., submitted). During the imaging, 13 fields were captured from each well, with two channels configured for ConA and DAPI, in 4 layers of z-stack. Raw.tiff images were processed using a custom Matlab script (Farkas et al., submitted) to select the optimal z-stack layer for each cell and to produce 696 × 520 pixels 8-bit.jpeg images (4 image per field of view), which were then used as inputs for the CalMorph software [43]. Quality control of raw morphological data and statistical analysis of processed morphological data were performed as described in (Farkas et al., submitted). Raw data of measurements and initial analysis in the form of boxplots were uploaded to FigShare (doi:10.6084/m9.figshare.15109488, 10.6084/m9.figshare.15105633 respectively).

*Pseudohyphal growth and flocculation*. Pseudohyphal growth was assessed after 10 days of growth at 37 °C by sampling colonies used for colony morphology and invasivity tests (described above). Samples of the colonies were viewed at 400× magnification with transmitted light. General morphology of pseudohyphae, if present, was recorded. Additionally, samples of the yeasts were pre-cultured overnight at 37 °C in YPD broth without shaking to observe potential flocculation in the medium. Furthermore, during epithelial transmigration experiments, as described below, yeast morphology was also recorded in the DMEM medium + 10% fetal bovine serum (FBS, non-USA origin, sterile-filtered, suitable for cell culture, Sigma-Aldrich) medium.

*High-throughput multicellularity measurement*. The Amnis FlowSight Imaging Flow Cytometer (Luminex, Austin, TX, USA) was used to investigate the presence of multicellular clumps and pseudohyphae among the isolates. This imaging flow cytometer has the advantage of recording a brightfield image of every event during standard flow cytometry. As a low and high aggregate forming control, the laboratory strains BY4743 and L5366 (respectively) were used. For this test, isolates were inoculated into YPD medium and grown until saturation in cell density at 25 °C with 140 rpm. The saturated cultures were diluted in PBS buffer to reach a cell density of 10^6^–10^7^ cells, and then the cell suspensions were immediately subjected to flow cytometry after vigorous shaking. The area (size of an event in square microns) and the aspect ratio (ratio of the minor axis divided by the major axis of an event) of the brightfield channel were estimated by the Amnis IDEAS software. Gating was applied based on the area parameter to exclude extrinsic noise (e.g., cellular debris). During each acquisition, a minimum of 5000 events were recorded. Area and aspect ratio values were exported and analyzed in R programming environment. As single round-shaped cells have both an aspect ratio around 1 and a low area value, we tested whether the isolates have a marked change in the above parameters, indicating more pronounced formation of pseudohyphae (or cell aggregates). As flocculation (clumps forming from cells not necessarily originating from the same cell) was absent in the isolates, events with larger areas and markedly changed aspect ratios were regarded as pseudohyphae (cell groups linked physically by incomplete separation after cell division). Analysis of multicellularity was performed on four independent samples and all measurements were pooled together. Area vs. aspect ratio plots and raw data were uploaded to FigShare (doi:10.6084/m9.figshare.15105633, 10.6084/m9.figshare.15109488, respectively).

### 2.4. Immune Tests

*Isolation and differentiation of human primary cells*. Peripheral blood mononuclear cells (PBMCs) were isolated by Ficoll–Paque (GE Healthcare, Uppsala, Sweden) density gradient centrifugation of heparinized leukocyte-enriched buffy coats of healthy donors drawn at the Regional Blood Center of Hungarian National Blood Transfusion Service (Debrecen, Hungary), with the written approval of the Director of the National Blood Transfusion Service and the Regional and Institutional Ethics Committee of the University of Debrecen, Faculty of Medicine (Debrecen, Hungary). Monocytes were purified from PBMCs by positive selection using anti-CD14-conjugated microbeads (Miltenyi Biotec, Bergish Gladbach, Germany), according to the manufacturer’s instructions. For dendritic cell (DC) differentiation, freshly isolated monocytes were seeded in 24 well cell culture plates at a density of 1 × 10^6^ cells/mL in RPMI 1640 medium (Sigma-Aldrich, St. Louis, MO, USA) supplemented with 10% heat-inactivated FBS (Life Technologies Corporation, Carlsbad, CA, USA), 2 mM L-glutamine, 100 U/mL penicillin, 100 mg/mL streptomycin (all from Sigma-Aldrich), 80 ng/mL granulocyte-macrophage colony stimulating factor (GM-CSF; Gentaur Molecular Products, London, UK) and 50 ng/mL interleukin-4 (IL-4; PeproTech, Brussels, Belgium) for 5 days. On day 2, the same amounts of GM-CSF and IL-4 were added to the cell cultures. The monocyte-derived DCs (moDC) were used for experiments on day 5 when more than 90% of the cells displayed immature DC phenotype (DC-SIGN/CD209+, CD14−). Allogenic CD3+ pan-T cells of the donors were isolated from PBMC using anti-CD3 microbeads (Miltenyi Biotec), according to the manufacturer’s instructions, and were used for moDC–T cell co-culture experiments as described below. All cells were incubated at 37 °C in 5% CO_2_ humidified atmosphere. Raw data of measurements for all immunological assays were uploaded to FigShare (doi:10.6084/m9.figshare.15145020).

*Phagocytosis assay.* Yeast cells were propagated overnight in YPD (30 °C, 140 rpm) before experiments. After three washing steps with PBS, yeast cells were resuspended in PBS and 1 × 10^8^ cell/mL were stained with 1 mg/mL FITC (fluorescein isothiocyanate, Sigma-Aldrich). The suspension was incubated in the dark for 30 min, at 37 °C, in the presence of 5% CO_2_. After incubation, excess FITC was washed from the yeast cells in two washing steps with PBS, then moDCs and yeast cells were co-cultured in 1:5 ratio (5 × 10^5^ iDCs + 2.5 × 10^6^ yeast cells) in complete RPMI 1640 medium. After 1 h incubation either at 37 °C or at 4 °C (as negative control), phagocytosis of DCs was terminated by washing the cells with cold buffer and samples were fixed with 100 µL 4% paraformaldehyde (PFA, Sigma-Aldrich) immediately. Phagocytosis was measured using a BD FACS Calibur Flow Cytometer (Becton Dickinson, Franklin Lakes, NJ, USA) and data analysis was performed using FlowJo Software (Treestar, Ashland, OR, USA). The events from onboard processing were eliminated and the proportion of moDCs with phagocytosed yeast cells was recorded.

*Phenotypic and functional analysis of yeast-exposed moDCs.* MoDCs were co-incubated with unstained yeast cells at a ratio of 1:3 (5 × 10^5^ moDC + 1.5 × 10^6^ yeast cells) for 24 h at 37 °C to determine the changes in their cell surface protein expression (CD40, CD80, CD83, CD86, HLA-DQ) and cytokine and chemokine secretion profile (IL-6, IL-8, IL-12, IL-1β, TNFα). Cell surface protein expression of yeast-exposed moDCs was analyzed by multicolor flow cytometry using anti-CD40-FITC, anti-CD80-FITC, anti-CD86-PE, anti-HLA-DQ-PE, anti-CD83-PeCy5 fluorescently labeled monoclonal antibodies, and isotype matched control antibodies (all from BioLegend, San Diego, CA, USA). Fluorescence intensities were measured by BD FACS Calibur Flow Cytometer (Becton Dickinson) and analysis of data was performed by the FlowJo software. Concentrations of inflammatory cytokines and chemokine produced by moDCs after yeast exposure were measured by enzyme-linked immunosorbent assay (ELISA) from the supernatant of the cell cultures. All BD OptEIA human ELISA assay kits specific for the measured cytokines or chemokines were obtained from BD Biosciences (San Diego, CA, USA) and ELISA kits were used according to the manufacturer’s instructions. Absorbance measurements were obtained with a Synergy HT microplate reader (Bio-Tek Instruments, Winooski, VT, USA) at 450 nm. Results where the measured fluorescence in cytokine/chemokine production was negligible were excluded from comparisons. Excluded donors are marked red in the raw measurements dataset.

*T cell activation assay.* To examine the T cell activating capacity of yeast-exposed moDCs, an enzyme-linked immunospot (ELISPOT) assay was performed. Following yeast treatments, moDCs were washed twice with cell culture medium and then co-cultured with allogeneic CD3+ pan-T cells in RPMI 1640 medium (Sigma-Aldrich) supplemented with 10% heat-inactivated FBS (Life Technologies Corporation), 2 mM L-glutamine, 100 U/mL penicillin, 100 mg/mL streptomycin (all from Sigma-Aldrich) in the presence of 1 mg/mL anti-human CD3 mAb (BD Biosciences) at a ratio of 1:10 (1 × 10^5^ moDC + 1 × 10^6^ T cell) in 48 well tissue culture plates. After 4 days, the cells were washed twice with PBS and the ratio of IFN-γ and IL-17 producing T cells was measured using human IFN-γ and IL-17 ELISPOT kits (eBioscience, Vienna, Austria) according to the manufacturer’s instructions. After completing the assays, the ELISPOT plates were dried, and spots were read on an ImmunoScan analyzer using ImmunoSpot 4.0 software (Cellular Technology Ltd., Bonn, Germany). Results where T-cell controls were high, activation was not found, or where replicate spot numbers were too variable were excluded from comparisons. Excluded donors are marked red in the raw measurements dataset in FigShare (doi:10.6084/m9.figshare.15145020, where ELISPOT images are also available).

### 2.5. Human Epithelium Model Interactions

Adhesion of yeast cells on human gastrointestinal epithelium model. Caco-2 (colon adenocarcinoma) cell line was obtained from the European Collection of Cell Cultures (ECACC, No. 86010202). These cells were routinely cultured (passages 20–30) in high glucose (4.5 g/L) Dulbecco’s Modified Eagle’s Media (DMEM) supplemented with 3.7 g/L NaHCO3, 10% heat-inactivated fetal bovine serum (FBS, non-USA origin, sterile-filtered, suitable for cell culture, Sigma Aldrich), 1% (*v*/*v*) non-essential amino acids solution (Lonza, Basel, Switzerland), 100 IU/mL penicillin and 100 µg/mL streptomycin mix (Lonza) at 37 °C in a humidified incubator in atmosphere of 5% CO_2_ (shortened in the following as cell culture medium). The glutamine was supplemented by GlutaMax™ (Thermo Fisher). For adhesion assays, 1 × 10^5^ Caco-2 cells were seeded in cell culture medium on glass coverslips (pre-treated with rat tail derived collagen I. beforehand by overnight incubation at 4 °C; Gibco—Thermo Fisher, Waltham, MA, USA), in 24 well culture plates with the same medium and incubated for 2–3 days [46] until the coverslips were overgrown with a continuous layer. The cells were infected with 10^5^ yeast cells (suspended in serum free cell culture medium) in triplicates, followed by 1 h incubation at 37 °C under 5% CO_2_ atmosphere, then unadhered cells were washed away with 1 mL PBS three times. The cells on coverslips were then fixed with 4% paraformaldehyde and were stained with calcofluor white. Stained coverslips were viewed using an Olympus BD-40 microscope equipped with 40× phase contrast and with a 40× fluorescence objective with transmitted light and phase contrast and with fluorescent excitation (respectively) to view adherent yeast cells. For each sample, three viewfields were observed on each of the triplicate slides, area of viewfield was calculated, and adherent cell number for 1 mm^2^ was subsequently calculated based on each individual viewfield. As a positive control, the SC5314 *Candida albicans* type strain was used which is known to be adherent to Caco-2 epithelium models using this experimental setup. Raw data of measurements for all epithelial model assays were uploaded to FigShare (doi:10.6084/m9.figshare.15145020).

Damage assay on Caco-2 human epithelium model. 10^4^ cells/well Caco-2 epithelial cells were seeded into 96 well microplates and incubated for 7 days prior to infection in culture medium described above [47]. Three groups were formed on each plate. 18 wells were seeded only with Caco-2 cells, 18 wells were seeded with Caco-2 and infected with yeast cells and 18 were inoculated only with yeast cells. For infection, 1 × 10^5^ *S. cerevisiae* cells/mL were co-incubated with the epithelial cells for 24 h in cell culture medium without FBS at 37 °C and humidified 5% CO_2_ atmosphere. The cell culture medium was refreshed at the moment of the infection for the non-infected group of wells, too. After the 24 h of incubation, all cells were treated with 1 mg/mL amphotericin B for 1 h. The antifungal agent was removed, cells were washed and 0.1 mL of 0.5 mg/mL solution of 2-(4,5-dimethyl-2-thiazolyl)-3,5-diphenyl-2H-tetrazolium bromide (MTT salt dissolved in PBS) was added to each well. Microplates were incubated for 3 h at 37 °C, then the dye was removed and 0.1 mL of a solution of isopropanol: 1 M hydrochloride acid (25:1) was added to each well to dissolve the cells. The absorbance of the wells was measured at 570 nm and 690 nm. All the measurements were carried out with a Multiskan Go (Thermo Fisher) microplate reader. For all data sets, the A570–A690 subtraction was completed and the mean of yeast wells was further subtracted from the values of the wells with infected Caco-2 cells. Cell viability in wells of infected Caco-2 cells with this correction was expressed as the percentage of the mean absorbance values of the uninfected Caco-2 cells, which were incubated with cell culture medium for 24 h. As a pathogenic positive control we used the type strain of *C. albicans* SC5314.

Epithelial transmigration assay with Caco-2 human epithelium model. In order to assess whether probiotic yeasts can cross the epithelial barrier in vitro, we used Transwell (Corning, NY, USA) cell culture insert following Pérez-Torrado et al. [27]. To obtain polarized monolayers, 6 × 10^4^ Caco-2 cells of the same passage number were seeded into Transwell cell culture inserts (Corning Incorporated, Corning, NY, USA) with 8 μm pore size, 1 × 10^5^ pores per cm^2^ density and 0.33 cm^2^ area, polycarbonate membrane, and placed in 24 well plates. In all cases, the volume of the apical compartment was set to 200 μL and the basolateral was set to 1250 μL. Caco-2 monolayers were used for experiments after 14–21 days of initial seeding, when the transepithelial electrical resistance (TEER) reached >450 Ω cm^2^. Before the infection, strains grown overnight at 30 °C in YPD were washed with PBS and resuspended in the cell culture medium in 1 × 10^6^ cells/mL concentration and were put into the apical compartment and incubated at 37 °C in a humidified atmosphere of 5% CO_2_. As a positive control capable of invasion through the epithelium model [27], the *C. albicans* SC5314 strain was used. Furthermore, we confirmed that yeasts cells are able to cross the Transwell insert if it is not seeded with Caco-2 cells. The medium in the apical and basolateral compartments were changed daily without disturbing the developing yeast layer. The total amount of medium from the lower compartment was centrifuged at 10,000× *g* and diluted in ddH_2_O, after which samples were observed in a haemocytometer and plated onto YPD plates, incubated at 30 °C for 3 days and colonies were counted, colony forming unit (CFU) numbers were then calculated. For every strain, three parallel wells were carried out. The experiment was conducted until 144 h was reached after inoculation, when the Caco-2 monolayers were starting to deteriorate. The presence of a biofilm in the upper compartment was checked visually during each daily addition of fresh medium (whether yeasts were easily suspended) using a binocular microscope and 20× magnification, transmitted light, and a 2 µL sample from the upper compartment was viewed under 400× magnification with transmitted visible light each day to examine pseudohyphal morphology.

### 2.6. Galleria Mellonella Larva Infection Model

The *Galleria mellonella* larva pathogenicity model was used to assess the pathogenicity of the strains and isolates in vivo. Larvae were obtained in two batches (Chameleonfarm, Budapest, Hungary) and used within 5 days. We used 2 × 20 last-instar specimens for infection experiments for each strain or isolate, with 20–20 larvae from two different batches. Altogether 2 × 20 specimens were used as negative controls inoculated with PBS. 20 uninfected controls were also used. As a pathogenic positive control we used the type strain of *C. albicans* SC5314. Injection followed [48] with 10^6^ cells (harvested after overnight incubation, washed three times in PBS) in PBS. Larvae were starved for 24 h at 30 °C before experiments to avoid temperature shock-driven immune activation before inoculation [9]. Each batch of larvae was used in one single run of inoculation experiments. Survival of the larvae was followed for 96 h after inoculation with incubation at 37 °C. From dead larvae, yeasts were recovered by homogenizing the larvae in 1 mL PBS and plating 10 µL onto YPD medium supplemented with 100 µg/mL ampicillin. These plates were incubated for 3 days at 30 °C. Colonies formed were visually observed, cells in colonies were viewed under 400× magnification with transmitted visible light to search for potential samples differing from *S. cerevisiae*/*S. ‘boulardii’* morphology.

### 2.7. Statistics and Data Visualization

To compare phenotypic results across groups of yeast isolates (commercial, mycosis, non-mycosis), VassarStats [49] was used to perform one-way ANOVA followed by Tukey honestly significant difference (HSD) tests or pairwise t-tests where only two groups were compared (yeast groups in the case of immunological experiments). For immunological experiments, not only isolate groups, but donors were also compared. Kruskal–Wallis test [50] was applied to high-throughput single-cell data to compare isolate groups, with Bonferroni correction (as no comparisons showed significant differences, post-hoc pairwise multiple comparison tests were not applied). For all continuous measurements, isolate groups were compared using the above-mentioned methods, while isolates were compared to each other using principal component analysis (PCA) with or without pre-defined groups and by generating heatmaps and phenotypic clustering using ClustVis [51]. Phenotypic clustering and PCA were used to assign isolates to categories in global comparisons of individual isolates. Analysis of raw data obtained by high-throughput single-cell phenotyping and multicellularity measurement were performed in R programming environment [50]. In the case of single-cell measurements, the first six principal components were further analyzed by assessing differences among isolates for the traits with the highest PC loadings. Measurement values were illustrated in GraphPad Prism 9 (GraphPad Software, Inc., San Diego, CA, USA), and to supplement these, in Excel (Microsoft Co., Redmond, WA, USA). For *Galleria* infections, Kaplan–Meier statistics and survival curve illustrations were performed using StatsKingdom [52].

## 3. Results

### 3.1. S. ‘boulardii’ Isolates

Among the 14 *S. ‘boulardii’* isolates used in this study, 11 were previously characterized with multiple genotyping methods and were shown to be genetically uniform (Table 1, Supplementary File S1). The three new additional isolates from the Szeged clinics also showed identical patterns with our multiplex PCR method and, furthermore, showed identical profiles in multi-locus sequence typing (Supplementary File S1), confirming their origins as derived from probiotic yeasts. Based on the origin and the pathogenic potential of the isolates we grouped the isolates into three groups: commercial (PY0001, PY0002, PY0003, PY0004), mycosis causing (DE6507, DE35762), and non-mycosis clinical isolates (DE27020, DE3912, DE42533, DE42807, DE45866, 465/2018, 551/2018, 2251/2018).

### 3.2. Phenotypic Differences among Commercial, Non-Mycosis, and Mycosis Isolates of S. ‘boulardii’

To investigate phenotypic differences among the probiotic isolates, we first investigated colony morphology. All isolates showed identical smooth and white colonies after 10 days of incubation. However, colony senescence assays revealed altered phenotypes that were variable (Supplementary File S2) and highly complex mainly in the case of the mycosis isolates. Colonies of the yeast isolates were found to be non-invasive on agar regardless of medium type or temperature. The formation of mitochondrial petite mutants was generally low, ranging from 0 to 1.22% of colonies, without significant differences among isolate groups (Supplementary File S2). None of the isolates sporulated either at 25 °C or at 37 °C, but MAT locus typing confirm that all of them were homothallic (Supplementary File S1). All samples were furthermore non-killer and sensitive to killer toxins (type 1 and 2), and flocculation was absent. Growth of the isolates assessed by the spot-plate method was identical for all samples at 37 °C and at 39 °C, while at 42 °C, the commercial PY0001 and PY0002 isolates showed more intense growth than other samples, whereas the isolate 2251/2018 was unable to grow (Supplementary File S2).

### 3.3. Virulence Factors and Pathogenicity of Commercial, Non-Mycosis, and Mycosis Isolates of S. ‘boulardii’

**Figure 1:**
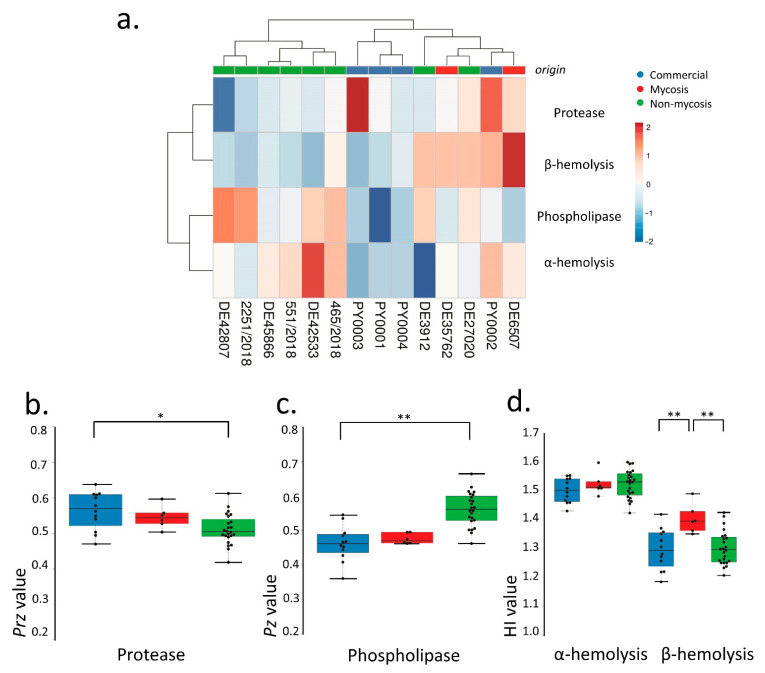
Comparisons of virulence factors (phospholipase and protease production, hemolysis): (**a**) Phenotypic clustering of isolates and heat map representation of results. Isolate groups are color-coded. Isolates are clustered using Euclidean distance and Ward linkage. For hemolysis, values after 2 d (α-hemolysis) and 3 d (β-hemolysis) were considered. (**b**–**d**) Box plot representation of virulence factor measurements for the isolate groups. (**b**) extracellular lipase *Pz* values; (**c**) extracellular protease *Prz* values; (**d**) haemolytic indices. Center lines show the medians; box limits indicate the 25th and 75th percentiles; whiskers extend 1.5 times the interquartile range from the 25th and 75th percentiles, outliers and data points are represented by dots. Comparisons with significant differences are marked (*: *p* < 0.05; **: *p* < 0.001).

To investigate virulence factor production of the isolates, we performed a series of assays established in clinical mycology research. We measured extracellular aspartate protease, lipase, and hemolytic activities (Figure 1). Phenotypic clustering separated the samples into two main groups, one having generally lower protease or β-hemolysis and higher phospholipase production, and one group with the opposite trends (Figure 1a). However, by examining these virulence factors separately, we found statistically significant differences between the groups. Tukey HSD test revealed significant difference between the commercial and the non-mycosis clinical isolates (*p* < 0.05) showing lower *Prz* (protease zone) values, thus higher protease production in the case of isolates that did not cause fungemia in the patients (Figure 1a). In case of the lipase production the mean of the measured *Pz*-values (phospholipase zone) was significantly higher (*p* < 0.01) in the case of the non-mycosis isolates, meaning that this group had the lowest lipase-producing ability (Figure 1b). The groups did not show significant difference in α-hemolysis, but the mycosis causing strains showed significantly higher (*p* < 0.01) β-hemolytic activity compared with the commercial and the non-mycosis clinical isolates (Figure 1c, Table 2, Supplementary File S2).

### 3.4. Cell Morphology and Pseudohypha Formation, Adhesion

**Figure 2:**
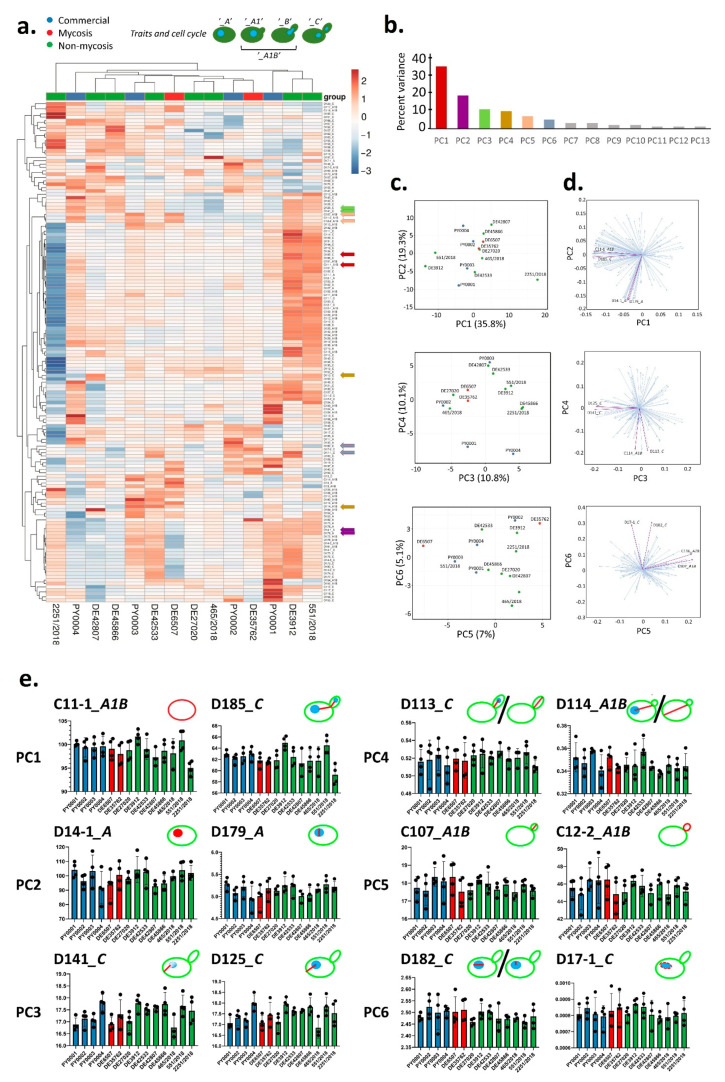
Evaluation of high-throughput single-cell phenotyping data. (**a**) Phenotypic clustering of measured traits, with unit variance scaling applied to rows. Rows are clustered using correlation distance and average linkage. Columns are clustered using Euclidean distance and average linkage. Trait groups according to cell cycle are explained in the form of schematic figures on top. Traits that were chosen to represent the first six principal components (PCs) are marked with arrows colored according to PC as in panel b. (**b**) Percentage of total variance explained by each PC, the six main PCs are colored as in panel a. (**c**) Results of principal component analysis, the six most important PCs are shown in pairwise plots. Isolates are colored according to isolate group. (**d**) PC loading plots in pairwise manner for the six most important PCs, with two representative traits highlighted (as in panel a). (**e**) Traits representing the six most important PCs (two traits for each PC, as in panels a and d). The means of measurements (bars) and individual measurements (dots) are shown for each isolate. Isolates are colored according to isolate group (commercial, mycosis, non-mycosis). Each trait is explained in the form of schematic figures where the measured feature is highlighted in red [C11-1: mother cell area. D185: total length of segment connecting the respective point on the outline of the mother cell intersected by the line connecting the mother cell nucleus center with the midpoint of the neck and the segment connecting the midpoint of the neck to the analogous point in the bud. D14-1: mother cell nucleus area. D179: nuclear minimum radius in mother. D141: distance between nuclear brightest point in mother and mother hip. D125: distance between nuclear gravity center in mother and mother hip. D113: distance between nuclear gravity center in bud and middle point of neck divided by long axis length in bud. D114: distance between nuclear gravity center and middle point of neck divided by distance between middle point of neck and mother hip. C107: long axis length in bud. C12-2: bud outline length. D182: ratio of mother nuclear long axis and nuclear minimum radius. D17-1: elliptical approximation of mother nucleus].

Phenotypic clustering of high throughput morphological data identified three groups of the isolates (Figure 2a) that mostly corresponded to the differences observed in traits most important for PC1 (responsible for 35.8% of total variance) in the principal component analysis (Figure 2b–d). The main traits contributing to PC1 were in relation to cell and bud size and roundness, those contributing to PC2 (responsible for 13.8% of total variance) were mostly related to nucleus dimensions (Figure 2b–d, Supplementary File S3). A single non-mycosis isolate, 2251/2018 was notably different from the rest of the yeast samples, with smaller, round cells but relatively large nucleus (Figure 2, Supplementary File S3). The non-mycosis isolates DE3912 and 551/2018 were characterized by larger, more elongated cells, the rest of the samples (all commercial, all mycosis, and the remaining non-mycosis yeasts) had values that placed them between these two morphological groups. None of the measured 149 traits showed significant differences when the yeast groups according to isolation source (commercial, mycosis and non-mycosis isolates) were compared (Supplementary File S3).

Pseudohyphal growth was observed to be medium-dependent (Supplementary File S2), and high-throughput analysis of pseudohyphae/aggregates identified a group with elevated (all commercial and mycosis isolates, and the non-mycosis 551/2018 isolate), one with medium (other non-mycosis isolates), and one with low pseudohypha formation. The latter group contained a single isolate, the above mentioned 2251/2018 (Supplementary File S3).

Adhesion to plastic surfaces was negligible in the case of all studied yeast, without significant differences among groups (Supplementary File S2).

### 3.5. Antifungal Susceptibility

Antifungal susceptibility testing showed higher caspofungin MIC values for the commercial yeasts when compared with the non-mycosis isolates. MIC values grouped the isolates into two clusters (Supplementary File S2). Values for Amphotericin B ranged from 0.125 to 0.25 μg/mL (the higher value was true for all but one isolate, namely PY0003). Values for fluconazole ranged from 2 to 8 μg/mL (half of the isolates showing 8 μg/mL), while caspofungin values were either 0.25 or 0.5.

### 3.6. Epithelial Interactions (Adhesion, Damage, Transmigration)

**Figure 3:**
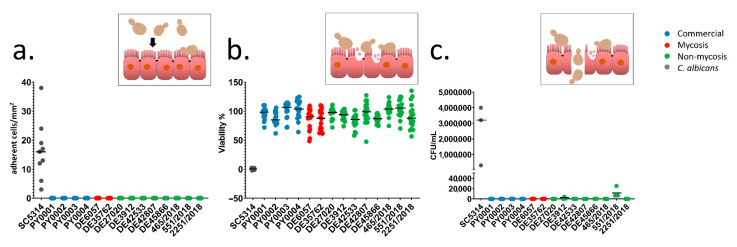
Interactions with epithelial model: adherence, damage, and transmigration. Inset pictures depict short explanations of the yeast-epithelium interactions (yeast cell sizes are exaggerated for better viewability). Individual data points are shown, colors represent yeast groups. Horizontal lines represent mean. (**a**) Adherence assay. Adherent yeast cells observed on Caco-2 epithelial model per mm^2^. (**b**) Damage assay. Caco-2 epithelial model relative viability (in %) after co-incubation with yeasts, based on MTT-assay. (**c**) Transwell assay. CFU/mL values in lower compartment of the Transwell plates are shown after co-incubating yeasts in the upper compartment with Caco-2 epithelial model on Transwell inserts for 144 h.

To investigate the adhesion and damage causing ability of the isolates to the intestinal epithelium, and the ability to cross through the epithelial barrier, the Caco-2 epithelium model was used. None of the yeast isolates adhered to the epithelial model and none of them caused damaged to it to an extent of more than 17% as measured with MTT assay (Figure 3a,b, Supplementary File S4). Transmission through the epithelium was not observable in the *S. ‘boulardii’* isolates, except from the non-mycosis isolates DE3912 and 551/2018, which could pass through the epithelium layer after four and six days, respectively (Figure 3c, Supplementary File S4). Biofilm formation was not observable even after six days of incubation during this experiment. The observable morphology in DMEM was mostly yeast and short pseudohyphae. In contrast to *S. ‘boulardii’*, *C. albicans*, used as a positive control, showed adhesion, damage, and rapid transmigration with hyphal growth in this model, as shown in Figure 3.

### 3.7. Immunological Interactions

**Figure 4:**
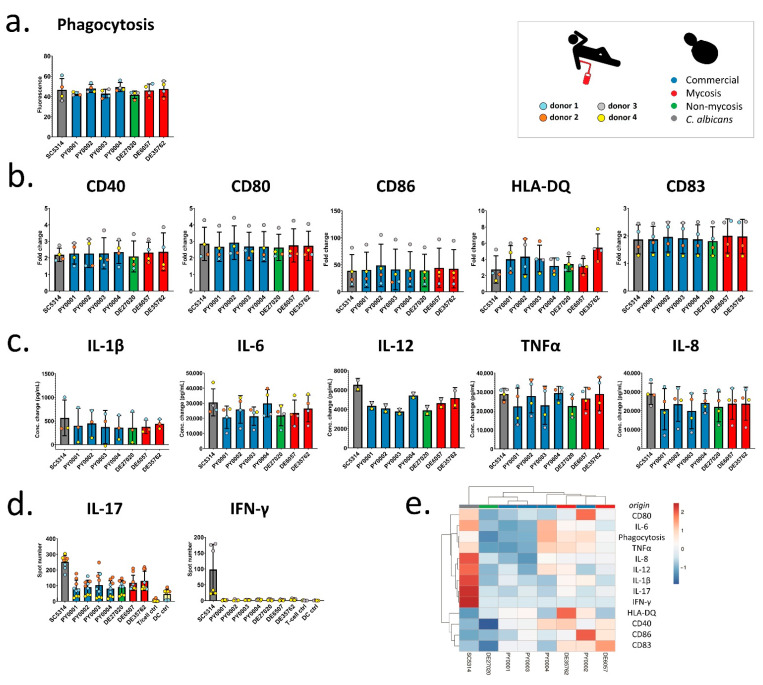
Immunological interactions. (**a**) Phagocytic activity of moDC for the various yeasts. Phagocytic activity of moDCs (fluorescence) corrected with uninfected and 4 °C controls shown for each donor. (**b**) Fold changes in measured moDC markers. Fold changes in fluorescence are depicted for moDC markers, except for CD83, where fold change in the percentage of positive cells is shown instead. (**c**) Cytokine/chemokine production. Change in concentration (pg/mL) shown for each measured cytokine/chemokine secretion. (**d**) T-cell activation by activated moDCs. Number of recorded spots in ELISPOT assay are shown. Photographs of ELISPOT assays are depicted in Supplementary File S7. (**a**–**d**): Bars represent mean values, whiskers show standard deviation. Individual data points are colored according to donor, bars are colored according to yeast group as shown in inset. (**e**) Phenotypic clustering of isolates based on immunological interactions. Unit variance scaling is applied to rows. Rows are clustered using correlation distance and average linkage. Columns are clustered using Euclidean distance and average linkage. Donor means were used for clustering.

Donor-dependent significant differences were not observed in the phagocytic activity of moDCs (Figure 4a), and, similarly, the commercial and mycosis groups did not differ significantly when phagocytic activity was compared (Table 3, Supplementary File S5). After co-incubation with yeast cells, we measured the amount of change of different cell surface molecules of DCs: costimulatory proteins (CD40, CD80, and CD86), the CD83 maturation marker, and the HLA-DQ antigen-presenting protein (Figure 4b). Fold changes in fluorescence (fold changes in percentage of positive DCs in the case of CD83) were compared to investigate the donor dependence effect, and we also compared the two groups of commercial and mycosis causing isolates. Except for HLA-DQ we found that the DCs from the different donors reacted variously when they were co-incubated with the yeast isolates, indicating that changes in the amount of cell surface molecules were highly dependent on the donor (Table 3, Figure 4b, Supplementary File S6). In addition, we did not find significant fold change differences between the commercial and mycosis causing isolates in any of the DC surface markers. Similarly, we determined the concentration changes of proinflammatory cytokines (IL-6, IL-12, IL-1β, TNFα) and IL-8 chemokine in the supernatant of the cells (Figure 4c). We found that the results were significantly different between the DCs obtained from individual donors, while significant differences were not found when comparing the commercial and mycosis groups (Table 3, Supplementary File S6). Tests of T-cell activation and polarization revealed not merely donor-dependent differences, but also significantly (two-sample *t*-test, *p* < 0.05) higher induction of IL-17 production by the mycosis isolates when compared with commercial ones (Figure 4d). Overall, phenotypic clustering of isolates based on all immunological interactions (applied to donor means, Figure 4e) clearly differentiated the *C. albicans* control from *S. ‘boulardii’* and formed two clusters within the latter. The cluster with generally higher immune activation contained two commercial and the two mycosis isolates (Figure 4e).

### 3.8. Galleria Infections

**Figure 5:**
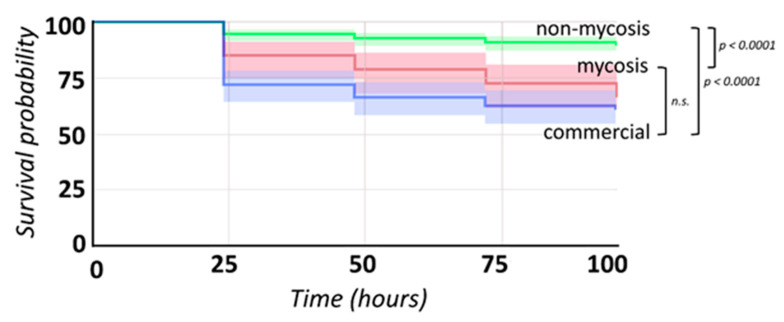
Survival curves of *Galleria* larvae inoculated with members of the commercial, non-mycosis, and mycosis sample groups. Significance of differences between groups is indicated on the right, based on Kaplan–Meier analysis, n.s. indicates that the difference is not significant. Shaded areas represent 95% log confidence interval.

Using the *G. mellonella* larva model, we compared the pathogenicity of all 14 isolates and the three isolate groups (commercial, non-mycosis, and mycosis) in this study, along with PBS and pathogenic *C. albicans* controls. The non-mycosis isolate group was altogether significantly less pathogenic than the mycosis or commercial group (Figure 5, Table 2) and within-group comparisons uncovered significant differences only in two cases, both involving the non-mycosis isolate 2251/2018 that was characterized by very high larval survival (Supplementary File S8).

## 4. Discussion

The existence of probiotic yeast derived infections prompted us to study virulence factors and host–microbe interactions following the methodology suggested by Anoop et al. [21]. Specifically, we measured classic virulence factors (phospholipase secretion, aspartate protease secretion, and hemolytic activity), thermotolerance, immune activation, adhesion to and interactions with the intestinal epithelium. We investigated additional phenotypic factors as well and used *Galleria melonella* larvae as animal model to investigate pathogenicity. Our experiments aimed to find potential phenotypic adaptations of *S. ‘boulardii’* in the human host that could be linked to increased survival, virulence, and overall, a pathogenic lifestyle. The phenotypic characteristics of this yeast and its interactions with cultured human epithelial or immune cells, pathogenic bacteria and yeasts, and with laboratory animals have been extensively studied. Its occasional pathogenicity in humans has been described by dozens of reports. But to our best knowledge, no studies have been conducted with the aim to characterize not just the strain or product itself, but also to compare it with isolates that potentially have evolved inside the human host (apart from our earlier study characterizing a single isolate [34]). Previous works have shown that the phenotypic characteristics of fungi do not merely determine their ability to colonize and invade a host but are also subjected to intense selection in the often hostile environment of the host’s various anatomical niches e.g., [53,54]. In particular, very few studies have been conducted on *S. cerevisiae* isolates [34,55,56], while most other works focused on other genera.

In our study, both the commercial and the clinical isolates originated from a single geographic region, east Hungary. All samples were void of extensive subculturing to prevent accumulation of mutations and genome structure variations during lab propagation. Furthermore, as clonal heterogeneity even in single batches of commercial yeasts may be prevalent [35], we used two different subclone isolates of each of the probiotic yeast products available in the country. Detailed patient and isolation data enabled us to compare non-mycosis and mycosis clinical samples as well.

Although the 14 isolates showed variability in phenotypic features (Supplementary File S2 and S3), in most cases, the commercial, the non-mycosis, and mycosis samples showed no difference in virulence factors (Figure 1, Table 2), adhesion to plastic, or in pseudohypha formation that are thought to be important in the steps of infection. Thermotolerance was highest for two commercial isolates. Notably, a single isolate (from the non-mycosis group), 2251/2018 was found to be distinct from the rest of the studied yeasts, showing altered cell morphology (smaller. more round cells with larger nucleus), low pseudohypha formation, and lower thermotolerance. Interestingly this isolate showed the lowest pathogenicity in the larva model, being significantly less pathogenic than nine other isolates. These observations point to decreased pathogenicity when compared to other human isolates, but notably also when compared to product isolates. This might indicate maladaptive traits or traits evolving towards a commensal lifestyle, but due to a lack of prolonged tracking of the gut microbiomes of the patients in this study, this question cannot be decided (compare with [34]).

Aspartate protease and lipase production as well as hemolytic activity are well known extracellular virulence factors in case of the opportunistic pathogen *Candida* species [57], yet, in our study, mycosis isolates did not show higher activities in most factors, only in β-hemolysis after 3 days. In the case of α-hemolysis measured after 1 day, the commercial isolates showed higher activity. Commercial isolates produced more phospholipase than the non-mycosis isolates, and the latter were more active in extracellular protease production (Table 2).

Antimycotic resistance assays showed high uniformity among the samples regarding amphotericin B and caspofungin susceptibility. For the former substance, the CLSI MIC Breakpoints for *Candida* species’ resistance are above 1 µg/mL, a value four times higher than the highest measured for our isolates. Caspofungin breakpoints for *Candida* species are highly variable, preventing us from comparing them with those of the *Saccharomyces* determined here. In the case of fluconazole, for most *Candida* species, resistant isolates have MIC values ≥8 µg/mL. This was reached by seven *Saccharomyces* isolates in our study, including both haemoculture samples.

In earlier studies with the Caco-2 epithelial model, it was shown that neither commercial nor clinical *S. cerevisiae* isolates could cross the epithelial barrier in vitro in 48 h, including an *S. ‘boulardii’* probiotic sample [23,27]. An additional report found low levels of traversal across the human endothelial barrier under similar in vitro conditions but did not use *S. ‘boulardii’* samples [58]. It must be noted that Klingberg et al. [23] proposed a link between the measured increase of trans-epithelial electric resistance (TEER) of polarized Caco-2 monolayers following exposure to *S. cerevisiae* strains during transmigration experiments and the strengthening of the epithelial barrier function, a potentially significant probiotic trait. However, our observations during the same type of experiments point to the fact that this is simply due to the accumulating but non-adherent yeast cell mass covering the epithelial model, which is easily disturbed during medium change and therefore cannot be linked to any effects (whether pathogenic or beneficial) on an in vivo gut epithelium. The isolates tested in our study showed negligible pathogenic interactions with the epithelium overall (Figure 3). However, interestingly, the only two isolates (DE3912, 551/2018 of the non-mycosis group) capable of crossing the epithelium in 5–6 days were the ones that showed markedly different, larger cells in single-cell phenotyping measurements. The potential relevance of this to pathogenicity is, however, hard to evaluate as the co-incubation was substantially prolonged before the transmigration was observed.

On the other hand, pathogenicity in the larva model showed, surprisingly, that the highest lethality was among larvae inoculated with the probiotic products, and not with the clinical samples (Figure 2, Supplementary File S8).

**Figure 6:**
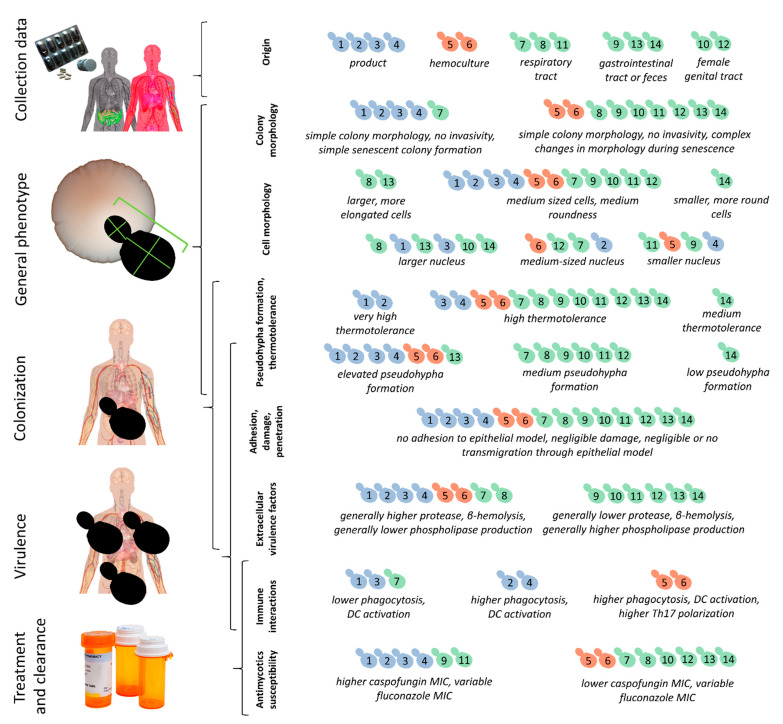
Summary of the clustering of *S. ‘boulardii’* isolates based on phenotypic differences, virulence factors, interactions with the epithelial model and with immune cells, and antimycotics susceptibility. Left: main steps and features of potential probiotic infections and probiotic yeasts in general. Right: individual isolates (blue: commercial, red: mycosis, green: non-mycosis) numbered according to Table 1, grouped into distinct categories based on simple comparisons, PCA, and phenotypic clustering.

Altogether, when PCA and phenotypic clustering were used to form phenotypically similar groups within the 14 isolates in this study (Figure 6), most groups contained commercial and clinical (mycosis and/or non-mycosis) isolates as well, there was no clear distinction based on origin. These results are summarized in Figure 6.

Regarding interactions with in vitro differentiated primary human dendritic cells and T-cells, the yeast isolates tested in our study displayed relatively small variation in their ability to elicit immune response or in the extent to which they were phagocytized by DCs, while donor-dependent differences in DC and T-cell activation were more pronounced, similar to our previous study with various *S. cerevisiae* clinical isolates [34] (Table 3, Figure 3, Supplementary Files S5–S7). Interactions with these actors of the innate and adaptive immune system are important not solely on the basis of the immune system’s role during a potential infection stemming from probiotic products, but also based on the fact that the yeast probiotic products themselves are known to exert their beneficial effects on the host by modulating the immune system. For example, anti-inflammatory properties are thought to be important in the case of *S. ‘boulardii’* as several studies have reported a reduction of intestinal inflammation through the modulation of DC function by these yeasts [59,60,61]. Yeast species are very much variable in their capacity to elicit inflammatory or pro-inflammatory responses from the (gut) immune system, even down to a strain-specific manner [34,61,62]. Immunogenicity of various yeasts correlate with the cell wall composition of strains [62] and it is unclear how immune modulation capability may change during commensal–pathogen transition in fungi. Differential recognition of specific cell wall mannan structures is crucial in the discrimination between harmful (e.g., *C. albicans*) and harmless yeasts (e.g., as generally recognized, *S. ‘boulardii’*) [63]. Evolving a pathogenic lifestyle from a commensal or probiotic strain, while retaining phenotypic characteristics that enables recognition as ‘harmless’ by the immune system is arguably an efficient evolutionary strategy, and this may be the case for the fungemia isolates in this study. In contrast with the other factors tested here, however, the Th-17 cell polarization found in the case of the two mycosis isolates may point to more inflammatory properties than that of the product isolates.

**Figure 7:**
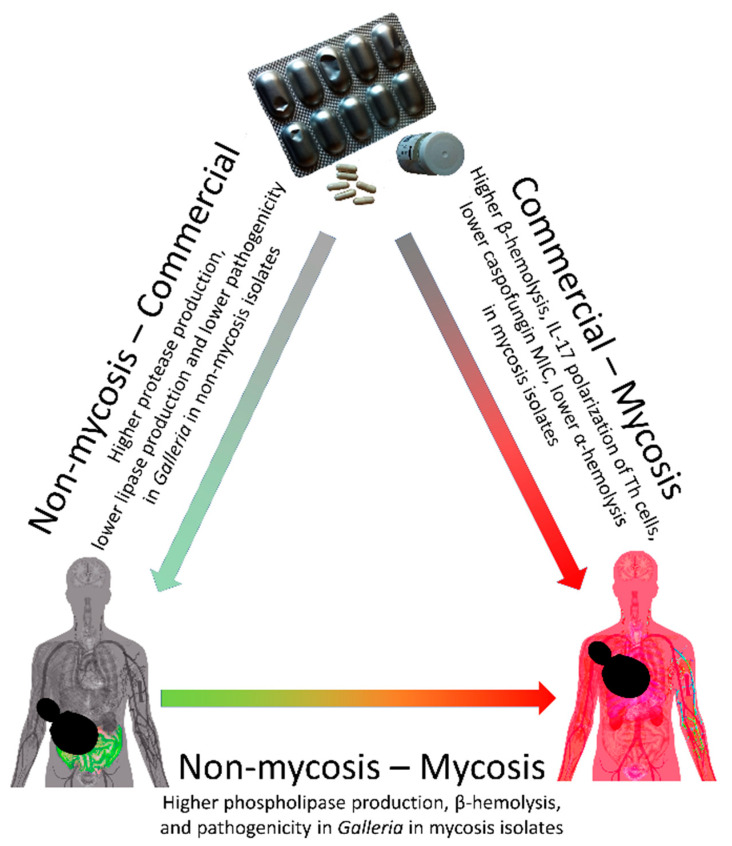
In this study we found variable phenotypic adaptations among *Saccharomyces* ‘*boulardii’*. Significant differences in product, non-mycosis, and mycosis isolates in phenotypic characteristics, antifungal sensitivity, virulence factors, immune interactions, and pathogenicity in larval model are described for each group-level comparison.

An intriguing finding of our study is that probiotic isolates that cause mycosis did not show such phenotypic traits that would enable them to actively invade the vascular system. Thus, the mechanism of entering the bloodstream and thereby causing systemic infection by probiotic isolates is still unclear. Nevertheless, previous studies have already proposed several possibilities in this regard. For example, it has been demonstrated that entry of yeast probiotic—besides the normal oral route—into the human body is possible through air [64,65], or from person to person [66]. Development of fungemia is possible due to catheter usage [65], intentional injection [67], and a case suggests that it is possible through other injuries in the gut resulting from gastrointestinal surgery [68]. Immunosuppressive treatments and conditions also predispose someone to fungal infections and its combination with the above-mentioned scenarios increase the risk of fungemia. Thus, yeast probiotics are contraindicated for patients with such serious health conditions. Despite these findings, in many cases these products are administered intentionally or unintentionally to patients with severe health conditions [14,15]. For instance, *S. ‘boulardii’* probiotics are applied most of the time in the presence of gastrointestinal dysbiosis, a condition that itself carries a risk of impaired barrier function embedding for additional infections [69,70,71]. The notion that yeast probiotic products may be hazardous in specific instances has already led one hospital to recently remove *S. ‘boulardii’* probiotic products from its pharmacy [17]. These and our results presented here thus support the idea that the entry of yeast probiotics into the bloodstream is not due to active mechanisms of the yeast itself but depends on the actual condition of the body. Survival and reproduction of these probiotic yeast strains in the bloodstream or in other niches in the human body might be attributed to several factors in the yeast. Future works should elucidate the exact molecular mechanisms underlying this phenotypic change during the transition from being a probiotic product/commensal yeast to having a pathogenic lifestyle. The differences in product, commensal (non-mycosis), and mycosis isolates in phenotypic characteristics, immune interactions, and other aspects tested in this study are summarized in Figure 7.

## 5. Conclusions

Selection in the host may result in population bottlenecks for probiotic and infectious yeast and novel geno- and phenotypes may emerge during in-host microevolution. We analyzed the phenotypic changes, changes in interactions with the epithelium, and differences in immune activation using 14 *S. ‘boulardii’* isolates. Four of these were product isolates, while ten were clinical samples already subjected to in-host selection. We found that in both groups (commercial and clinical), individual yeast isolates may show different phenotypes, but clear differentiation between commercial and clinical, or clinical mycosis and clinical non-mycosis samples is not apparent. This shows that *S. ‘boulardii’*, a popular probiotic microbe investigated by hundreds of studies for its effects on health, described as a potential pathogen by many dozen studies, and regularly taken by millions of people, can infect or survive in the human host for extended time periods without evolving characteristics that earlier studies aimed to link with pathogenicity. In other words, currently screened virulence factors fail to differentiate among virulent mycosis isolates, isolates virulent in animal models, and the isolates from generally regarded as safe probiotic products that have not yet been subjected to in-host selection in the first place. The resistance to an antifungal agent (fluconazole) in probiotic products is also noteworthy.

Given the results reported in the present and previous studies, the use of yeast products should be regulated and controlled more carefully in the future. In addition, further research should be conducted to explore the properties of yeast that allow it to survive in the various niches of the human body, but with a greater emphasis on the use of animal models instead of measuring virulence factors established mainly in, and applied from, the *Candida* literature.

## Figures and Tables

**Table 1:** Collection and patient data for the isolates used in this study.

ID	Isolate	Type	Formulation		Component		Place of Acquisition	Date of Acquisition	Country of Manufacturing	Reference
1	PY0001	probiotic supplement	active dry		single component		Debrecen, Hungary	March 2015	France	[16,34]
2	PY0002	probiotic supplement	active dry		single component		Debrecen, Hungary	November 2017	France	[16]
3	PY0003	probiotic supplement	active dry		multicomponent		Debrecen, Hungary	September 2017	Czechia	[16]
4	PY0004	probiotic supplement	active dry		multicomponent		Debrecen, Hungary	November 2017	Czechia	[16]
**ID**	**Isolate**	**Type**	**Age (yr) at Sampling**	**Sex**	**Prevailing Medical Condition during Isolation**	**Mycosis Case**	**Anatomical Origin/Sample Type**	**Date of Sampling**	**Geographic Origin**	**Reference**
5	DE6507	clinical isolate (probiotic-derived)	63	♂	pneumonia	yes	haemoculture	18 February 2017	Debrecen, University Clinic	[16]
6	DE35762	clinical isolate (probiotic-derived)	66	♀	respiratory failure	yes	haemoculture	5 November 2015	Debrecen, University Clinic	[16]
7	DE27020	clinical isolate (probiotic-derived)	40	♀	sepsis (bacterial)	no	bronchus (sampling during intubation)	23 August 2015	Debrecen, University Clinic	[16,34]
8	DE3912	clinical isolate (probiotic-derived)	85	♂	pneumonia	no	trachea (sampling from tracheal cannula)	31 January 2018	Debrecen, University Clinic	[16]
9	DE42533	clinical isolate (probiotic-derived)	2	♂	fluid homeostasis disorder	no	throat	15 December 2017	Debrecen, University Clinic	[16]
10	DE42807	clinical isolate (probiotic-derived)	1	♀	diarrhea	no	vagina	4 December 2017	Debrecen, University Clinic	[16]
11	DE45866	clinical isolate (probiotic-derived)	64	♂	cerebral infarction	no	bronchus (sampling during intubation)	29 December 2017	Debrecen, University Clinic	[16]
12	465/2018	clinical isolate (probiotic-derived)	41	♀	amenorrhea	no	vagina	3 January 2018	Szeged, University Clinic	-
13	551/2018	clinical isolate (probiotic-derived)	81	♂	paralytic ileus	no	feces	3 January 2018	Szeged, University Clinic	-
14	2251/2018	clinical isolate (probiotic-derived)	17	♂	ulcerative colitis	no	feces	8 January 2018	Szeged, University Clinic	-

**Table 2:** Statistical comparison of isolate groups for measurable phenotypes and for *Galleria* experimental infections. Statistical results for single-cell phenotyping are shown in Supplementary File S3.

	Biofilm Formation on Plastic Surface	Secreted Enzymatic Virulence Factors		Hemolytic Index, 37 °C				*Galleria* Larva Survival	
	YPD Liquid, 37 °C, 3 d	Phospholipase Secretion, *Pz* Value, 37 °C	Aspartate Protease Secretion, *Prz* Value, 37 °C	α-Hemolysis, 1 d	α-Hemolysis, 2 d	β-Hemolysis, 2 d	β-Hemolysis, 3 d	*p* Value Log-Rank Test	
ANOVA *p* Value	0.53792	<0.0001	0.00577	0.03899	0.36651	0.91412	0.00182		
Commercial vs. Mycosis (Tukey HSD)	n.s. (*p* ≥ 0.05)	n.s. (*p* ≥ 0.05)	n.s. (*p* ≥ 0.05)	C > M (*p* < 0.05)	n.s. (*p* ≥ 0.05)	n.s. (*p* ≥ 0.05)	C < M (*p* < 0.01)	Commercial vs. Mycosis	0.30283
Commercial vs. Non-mycosis (Tukey HSD)	n.s. (*p* ≥ 0.05)	C < NM (*p* < 0.01)	C > NM (*p* < 0.05)	n.s. (*p* ≥ 0.05)	n.s. (*p* ≥ 0.05)	n.s. (*p* ≥ 0.05)	n.s. (*p* ≥ 0.05)	Commercial vs. Non-mycosis	<0.0001
Mycosis vs. Non-mycosis (Tukey HSD)	n.s. (*p* ≥ 0.05)	M < NM (*p* < 0.01)	n.s. (*p* ≥ 0.05)	n.s. (*p* ≥ 0.05)	n.s. (*p* ≥ 0.05)	n.s. (*p* ≥ 0.05)	M > NM (*p* < 0.01)	Mycosis vs. Non-mycosis	<0.0001

**Table 3:** Statistical comparison of isolate groups and donors for DC and T-cell activation, along with DC phagocytosis.

	Trait	Phagocytosis	CD40 (Costimulatory)	CD80 (Costimulatory)	CD86 (Costimulatory)	CD83 (Maturation Marker)	HLA- DQ (Antigen-Presenting)	IL-6 (Proinflammatory)	IL-8 (Chemokine)	TNFα (Proinflammatory)	IL-12 (Proinflammatory)	IL-1β (Proinflammatory)	IL-17 (Proinflammatory)	IFN-γ (Proinflammatory)
Donor-dependence	ANOV A/T-test *p*-value	0.10721	<0.00010	<0.0001	<0.0001	<0.0001	0.54060	<0.0001	<0.0001	<0.0001	0.07714 (T-test)	<0.0001	<0.0001	0.00026 (T-test)
	Tukey HSD/T-test	none	D1 < D3 (*p* < 0.01)	D1 < D3 (*p* < 0.01)	D1 < D2 (*p* < 0.01)	D1 > D2	none	D1 > D3	D1 > D2 (*p* < 0.05)	D1 > D3 (*p* < 0.01)	none (T-test)	D1 > D2 (*p* < 0.01)	D1 < D2 (*p* < 0.01)	D3 > D4 (T-test)
			D2 < D3 (*p* < 0.01)	D1 < D4 (*p* < 0.01)	D1 < D3 (*p* < 0.01)	D1 < D3 (*p* < 0.0)		D2 > D3 (*p* < 0.0)	D1 > D3 (*p* < 0.01)	D1 > D4 (*p* < 0.01)		D1 > D4 (*p* < 0.01)	D1 > D4 (*p* < 0.01)	
			D3 > D4 (*p* < 0.01)	D2 < D3 (*p* < 0.01)	D1 < D4 (*p* < 0.01)	D1 > D4		D3 < D 4	D1 > D4 (*p* < 0.01)	D2 > D3 (*p* < 0.01)		D2 > D4 (*p* < 0.01)	D2 > D4 (*p* < 0.01)	
				D2 < D4 (*p* < 0.01)	D2 < D3 (*p* < 0.01)	D2 < D3			D2 > D3 (*p* < 0.01)	D2 > D4 (*p* < 0.01)				
				D3 > D4 (*p* < 0.01)	D2 < D4 (*p* < 0.01)	D2 > D4 (*p* < 0.0)			D3 < D4 (*p* < 0.01)					
					D3 > D4 (*p* < 0.01)	D3 > D4								
Commercial vs. Mycosis	T-test	0.64298	0.87434	1.0000	0.98422	0.70758	0.56780	0.88993	0.65003	0.54811	0.31408	0.89588	0.03792 (C < M)	0.11320

## Data Availability

The data underlying this article are available in FigShare at https://doi.org/10.6084/m9.figshare.15145020 (accessed on 8 September 2021) (raw phenotyping results, ELSIPOT images), and 10.6084/m9.figshare.15105633 (high throughput microscopy, FlowSight).

## Supplementary Materials

The following are available online at www.mdpi.com/2309-608X/7/9/746/s1. Supplementary File S1: Genotyping results; Supplementary File S2: Phenotyping results; Supplementary File S3: High-throughput phenotyping results; Supplementary File S4: Epithelium interactions; Supplementary File S5: Interactions with primary dendritic cells: phagocytic activity; Supplementary File S6: Interactions with primary dendritic cells: activation; Supplementary File S7: Primary T-cell activation by activated dendritic cells; Supplementary File S8: *Galleria* larva pathogenicity model.
