# Supplementary material for: Virulence Factors and in-Host Selection on Phenotypes in Infectious Probiotic Yeast Isolates (Saccharomyces ‘boulardii’)
Source: J Fungi (Basel). 2021 Sep 11;7(9):746. doi: 10.3390/jof7090746 (PMC8472476; doi:10.3390/jof7090746)

Supplementary Material

Virulence factors and in-host selection on phenotypes in infectious probiotic yeast isolates (*Saccharomyces 'boulardii'*)

Supplementary File S1. Genotyping results. 2

Supplementary File S2. Phenotyping results.................... 4

Supplementary File S3. High-throughput phenotyping results 9

Supplementary File S4. Epithelium interactions 15

Supplementary File S5. Interactions with primary dendritic cells: phagocytic activity 18

Supplementary File S6. Interactions with primary dendritic cells: activation 19

Supplementary File S7. Primary T-cell activation by activated dendritic cells 22

Supplementary File S8. *Galleria* larva pathogenicity model 24

Supplementary File S1.

Genotyping results.

Sample		Multiplex PCR genotyping	Multi Locus Sequence Typing (MLST)					MAT locus typing
			GenBank IDs for MLST gene sequences				% difference in alignment	
Species	Isolate	Position on gel image	*CCA1* (chr. V) sequenced: 526-1313	*CYT1* (XV) sequenced: 1-930	*HMX1* (chr. XII) sequenced: 1-954	*NUP116* (XIII) sequenced: 2321-2747		
Saccharomyces cerevisiae	PY0001	1	MK514561	MK514568	MK514575	MK514582	0%	a/α
Saccharomyces cerevisiae	PY0002	2	MK514562	MK514569	MK514576	MK514583	0%	a/α
Saccharomyces cerevisiae	PY0003	3	MK514563	MK514570	MK514577	MK514584	0%	a/α
Saccharomyces cerevisiae	PY0004	4	MK514564	MK514571	MK514578	MK514585	0%	a/α
Saccharomyces cerevisiae	DE27020	5	MK514565	MK514572	MK514579	MK514586	0%	a/α
Saccharomyces cerevisiae	DE6057	6	MK514566	MK514573	MK514581	MK514587	0%	a/α
Saccharomyces cerevisiae	DE35762	7	MK514567	MK514574	MK514580	MK514588	0%	a/α
Saccharomyces cerevisiae	DE3912	8	MK993596	MK993608	MK993630	MK993620	0%	a/α
Saccharomyces cerevisiae	DE42533	9	MK993597	MK993609	MK993631	MK993621	0%	a/α
Saccharomyces cerevisiae	DE42807	10	MK993598	MK993610	MK993632	MK993622	0%	a/α
Saccharomyces cerevisiae	DE45866	11	MK993599	MK993611	MK993633	MK993623	0%	a/α
Saccharomyces cerevisiae	465/2018	12	MZ712202	MZ712205	MZ712208	MZ712211	0%	a/α
Saccharomyces cerevisiae	551/2018	13	MZ712203	MZ712206	MZ712209	MZ712212	0%	a/α
Saccharomyces cerevisiae	2251/2018	14	MZ712204	MZ712207	MZ712210	MZ712213	0%	a/α

Gel image of Multiplex PCR method applied to identify *S. 'boulardii'*. M: 1 kb size marker. n: negative (no template DNA) control. Isolate numbers as above in table.

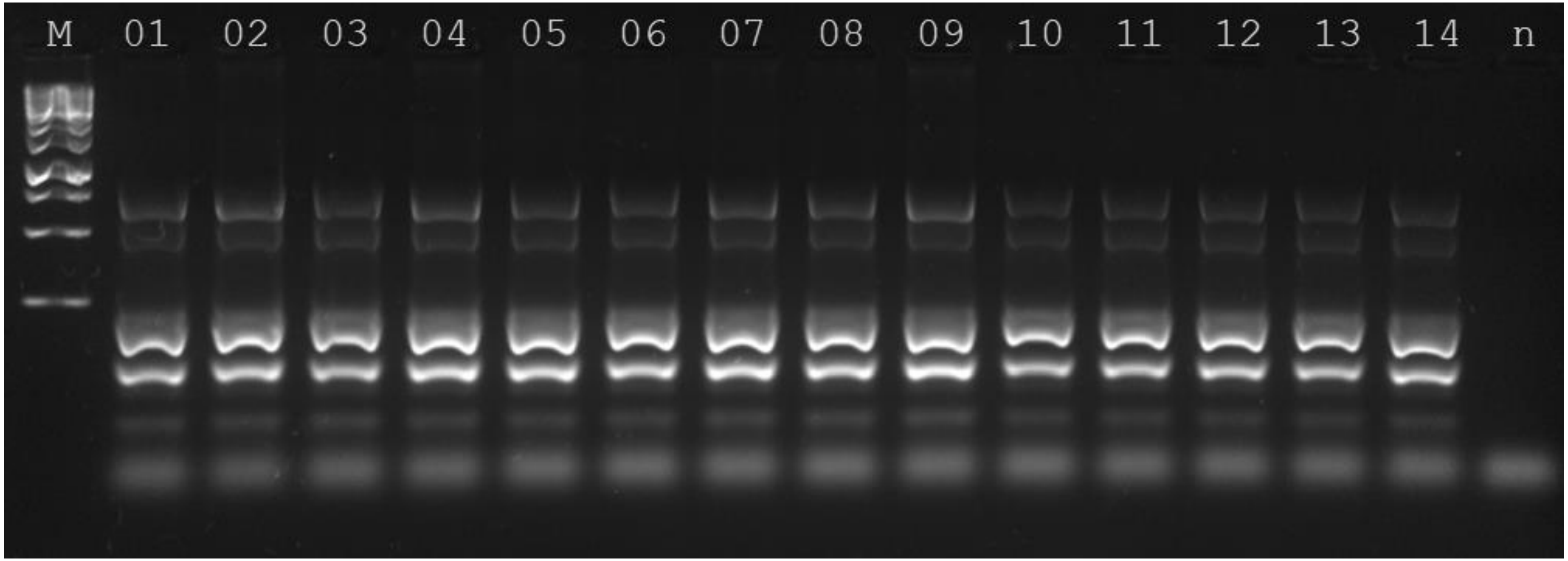

Supplementary File S2.

General phenotyping results for the *S. 'boulardii'* isolates.

Isolate	Colony morphology		Killer activity			Sporulation efficiency (%)		Petite frequency, GlyYP 30°C 10 d
	YPD 37°C 10 d	GlyYP 37°C 30 d (senescence)	Killer toxin 1 sensitivity	Killer toxin 2 sensitivity	Killer toxin production	K-Ac 37°C 10 d	K-Ac 25°C 10 d	
PY0001	smooth, white	simple: smooth, slightly sectored, white	yes	yes	no	0%	0%	0.29%
PY0002	smooth, white	simple: smooth, slightly sectored, white	yes	yes	no	0%	0%	0%
PY0003	smooth, white	complex: rough, moderately sectored, white	yes	yes	no	0%	0%	0.31%
PY0004	smooth, white	complex: rough, slightly sectored, white	yes	yes	no	0%	0%	0.30%
DE6507	smooth, white	complex: rough, highly sectored with large sub-colonies coming from sectors, white	yes	yes	no	0%	0%	0.33%
DE35762	smooth, white	complex: extensively rough, sectored, yellow-brown	yes	yes	no	0%	0%	0.86%
DE27020	smooth, white	simple: smooth, moderately sectored, white	yes	yes	no	0%	0%	0.27%
DE3912	smooth, white	complex: rough, slightly sectored, white	yes	yes	no	0%	0%	0.31%
DE42533	smooth, white	complex: rough, slightly sectored, white	yes	yes	no	0%	0%	1.03%
DE42807	smooth, white	complex: rough, slightly sectored, white	yes	yes	no	0%	0%	0.84%
DE45866	smooth, white	complex: rough, slightly sectored, white	yes	yes	no	0%	0%	1.57%
465/2018	smooth, white	complex: smooth, moderately sectored, few rough colony areas, white	yes	yes	no	0%	0%	0.94%
551/2018	smooth, white	complex: extensively rough, sectored, yellow-brown	yes	yes	no	0%	0%	0.39%
2251/2018	smooth, white	complex: smooth, highly sectored, white	yes	yes	no	0%	0%	1.11%

Isolate	Growth at high temperatures			Invasivity on agar media				Flocculation liquid medium	Pseudohyphal growth in liquid medium (type and abundance of pseudohyphae)		Biofilm formation on plastic surfaces (number of adherent cell patches per sq. mm)
	37°C	39°C	42°C	YPD 37°C 10 d	YPD 39°C 2 d	SLAD 37°C 10 d	SLG 37°C 10d	YPD liquid 37°C 1–3 d	YPD liquid + 10% FBS 37°C 1 d	DMEM+10% FBS 37°C 1–10 d in Caco-2 transmigration assay	YPD liquid, cell culture treated polystyrene plates, 37°C, 3 d
PY0001	+++	++	+	no	no	no	no	no	short, mostly pseudohyphae	short, mostly pseudohyphae	0 (SD: 0.00)
PY0002	+++	++	+	no	no	no	no	no	short, mostly pseudohyphae	short, mostly pseudohyphae	4.29 (SD: 5.11)
PY0003	+++	++	+-	no	no	no	no	no	short, mostly pseudohyphae	short, mostly pseudohyphae	1.30 (SD: 2.26)
PY0004	+++	++	+-	no	no	no	no	no	short, mostly pseudohyphae	short, mostly pseudohyphae	1.51 (SD: 2.62)
DE6507	+++	++	+-	no	no	no	no	no	short, mostly pseudohyphae	short, mostly pseudohyphae	0 (SD: 0.00)
DE35762	+++	++	+-	no	no	no	no	no	short, mostly pseudohyphae	short, mostly pseudohyphae	0.53 (SD: 0.92)
DE27020	+++	++	+-	no	no	no	no	no	short, mostly pseudohyphae	short, mostly pseudohyphae	0 (SD: 0.00)
DE3912	+++	++	+-	no	no	no	no	no	short, mostly pseudohyphae	short, mostly pseudohyphae	0.11 (SD: 0.20)
DE42533	+++	++	+-	no	no	no	no	no	short, mostly unicellular	short, mostly pseudohyphae	0 (SD: 0.00)
DE42807	+++	++	+-	no	no	no	no	no	short, mostly unicellular	short, mostly pseudohyphae	0.30 (SD: 0.52)
DE45866	+++	++	+-	no	no	no	no	no	short, mostly unicellular	short, mostly pseudohyphae	0.25 (SD:0.43)
465/2018	+++	++	+-	no	no	no	no	no	short, mostly unicellular	short, mostly pseudohyphae	3.99 (SD: 5.20)
551/2018	+++	++	+-	no	no	no	no	no	short, almost exclusively unicellular	short, mostly pseudohyphae	3.57 (SD: 6.19)
2251/2018	+++	++	-	no	no	no	no	no	no	short, mostly pseudohyphae	5.95 (SD: 1.73)

Isolate	Antimycotic susceptibility			Secreted enzymatic virulence factors		Hemolytic index, 37°C			
	Ampho-tericin B MIC values, µg/mL	Fluconazole MIC values, µg/mL	Caspofungin MIC values, µg/mL	Phospho-lipase secretion, Pz value, 37°C	Aspartate protease secretion, Prz value, 37°C	α-hemolysis, 1 d	α-hemolysis, 2 d	β-hemolysis, 2 d	β-hemolysis, 3 d
PY0001	0.25	8	0.5	0.40 (SD: 0.03)	0.53 (SD: 0.03)	1.16 (SD: 0.07)	1.49 (SD: 0.05)	1.29 (SD: 0.08)	1.27 (SD: 0.08)
PY0002	0.25	8	0.5	0.51 (SD: 0.05)	0.60 (SD: 0.02)	1.17 (SD: 0.03)	1.55 (SD: 0.01)	1.33 (SD: 0.05)	1.38 (SD: 0.03)
PY0003	0.125	4	0.5	0.47 (SD: 0.02)	0.62 (SD: 0.02)	1.29 (SD: 0.04)	1.48 (SD: 0.05)	1.29 (SD: 0.04)	1.25 (SD: 0.05)
PY0004	0.25	8	0.5	0.47 (SD: 0.02)	0.51 (SD: 0.04)	1.31 (SD: 0.06)	1.49 (SD: 0.03)	1.26 (SD: 0.04)	1.29 (SD: 0.04)
DE6507	0.25	8	0.25	0.47 (SD: 0.01)	0.56 (SD: 0.03)	1.17 (SD: 0.04)	1.53 (SD: 0.06)	1.31 (SD: 0.05)	1.44 (SD: 0.05)
DE35762	0.25	8	0.25	0.49 (SD: 0.02)	0.53 (SD: 0.02)	1.16 (SD: 0.03)	1.52 (SD: 0.01)	1.27 (SD: 0.03)	1.37 (SD: 0.02)
DE27020	0.25	4	0.25	0.55 (SD: 0.01)	0.55 (SD: 0.07)	1.16 (SD: 0.01)	1.51 (SD: 0.02)	1.31 (SD: 0.04)	1.37 (SD: 0.04)
DE3912	0.25	4	0.25	0.57 (SD: 0.04)	0.51 (SD: 0.06)	1.23 (SD: 0.06)	1.45 (SD: 0.02)	1.23 (SD: 0.06)	1.37 (SD: 0.07)
DE42533	0.25	4	0.5	0.57 (SD: 0.06)	0.51 (SD: 0.01)	1.12 (SD: 0.02)	1.58 (SD: 0.02)	1.35 (SD: 0.02)	1.25 (SD: 0.05)
DE42807	0.25	8	0.25	0.61 (SD: 0.06)	0.46 (SD: 0.04)	1.19 (SD: 0.03)	1.52 (SD: 0.05)	1.27 (SD: 0.03)	1.27 (SD: 0.05)
DE45866	0.25	2	0.5	0.50 (SD: 0.03)	0.51 (SD: 0.01)	1.22 (SD: 0.06)	1.53 (SD: 0.03)	1.30 (SD: 0.07)	1.28 (SD: 0.05)
465/2018	0.25	8	0.25	0.58 (SD: 0.02)	0.53 (SD: 0.02)	1.24 (SD: 0.08)	1.55 (SD: 0.02)	1.30 (SD: 0.03)	1.33 (SD: 0.02)
551/2018	0.25	4	0.25	0.51 (SD: 0.02)	0.52 (SD: 0.06)	1.18 (SD: 0.01)	1.54 (SD: 0.06)	1.29 (SD: 0.01)	1.27 (SD: 0.02)
2251/2018	0.25	4	0.25	0.60 (SD: 0.01)	0.50 (SD: 0.01)	1.14 (SD: 0.02)	1.50 (SD: 0.03)	1.23 (SD: 0.02)	1.26 (SD: 0.01)

Colony images of senescence (GlyYP 37°C 30 d). a: PY0001; b: PY0002; c: PY0003; d: PY0004; e: DE27020; f: DE6507; g: DE35762; h: DE3912; i: DE42533; j: DE42807; k: DE45866; l: 465/2018; m: 551/2018; n: 2251/2018. Three individual colonies photographed for each isolate.

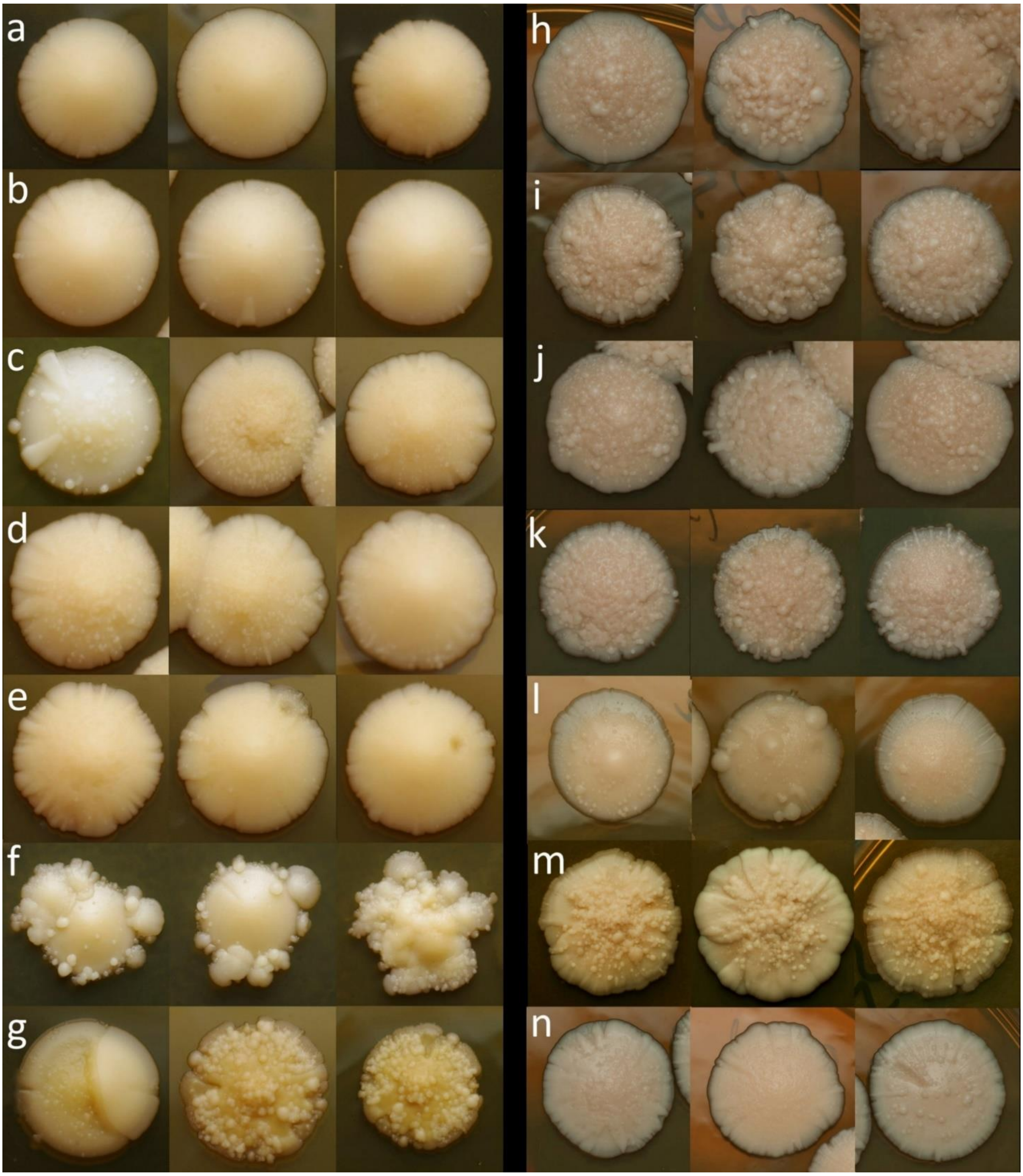

Phenotypic clustering of antimycotic MIC values. Isolate groups (origin) are color-coded. Unit variance scaling is applied to rows. Both rows and columns are clustered using correlation distance and average linkage.

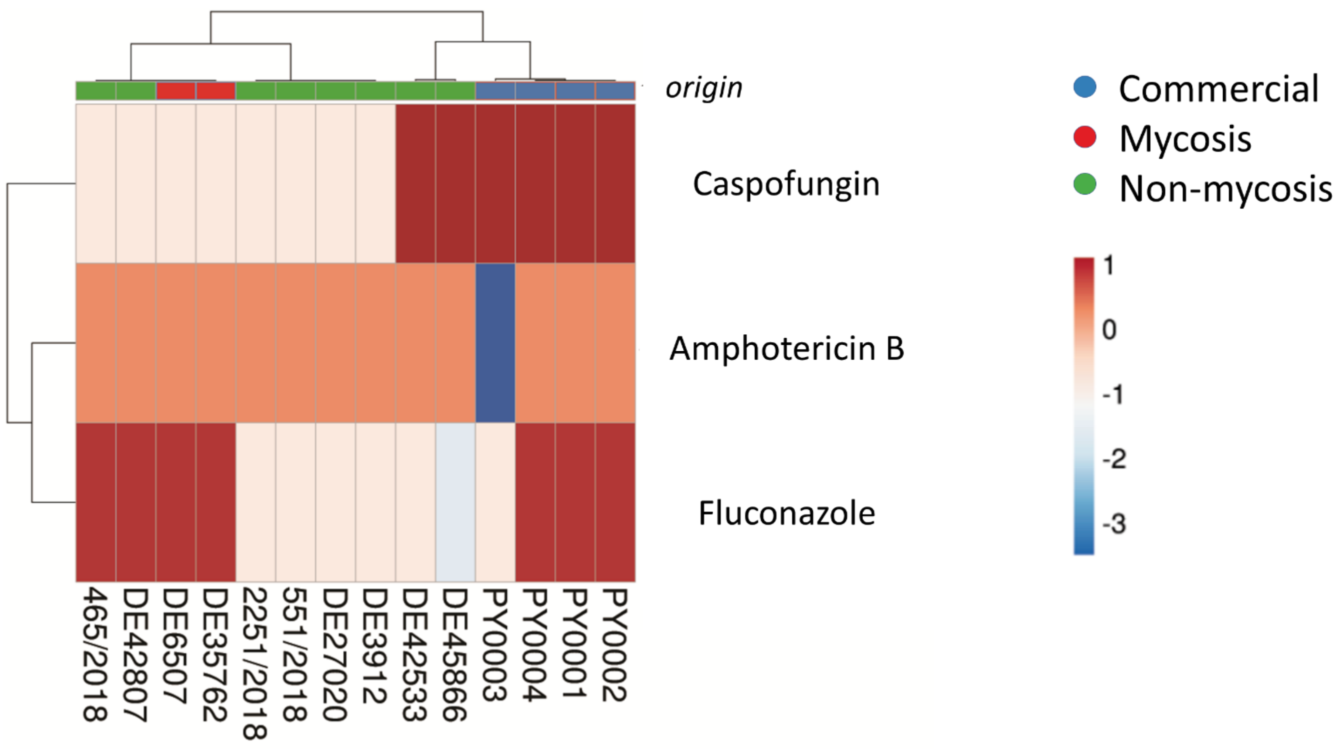

Supplementary File S3.

High-throughput phenotyping results.

Sample		FlowSight multicellularity analysis					
		Area			Aspect ratio		
Species	Strain/Isolate	Mean	S.D.	99.9% quantile value	Mean	S.D.	99.9% quantile value
Saccharomyces cerevisiae	BY4743	135.518	44.946	418.580	0.762	0.131	0.998
Saccharomyces cerevisiae	L5366	158.308	61.802	530.000	0.743	0.130	0.997
Saccharomyces cerevisiae	PY0001	137.271	49.515	391.672	0.743	0.146	0.997
Saccharomyces cerevisiae	PY0002	135.678	48.245	409.397	0.741	0.145	0.994
Saccharomyces cerevisiae	PY0003	135.173	47.882	398.865	0.739	0.146	0.995
Saccharomyces cerevisiae	PY0004	135.078	47.558	391.586	0.738	0.145	0.996
Saccharomyces cerevisiae	DE6057	132.374	45.574	388.147	0.740	0.143	0.993
Saccharomyces cerevisiae	DE35762	133.738	46.317	382.000	0.739	0.145	0.995
Saccharomyces cerevisiae	DE27020	136.316	48.756	403.750	0.724	0.145	0.993
Saccharomyces cerevisiae	DE3912	134.009	49.449	406.000	0.724	0.143	0.992
Saccharomyces cerevisiae	DE42533	133.764	46.860	388.092	0.730	0.143	0.995
Saccharomyces cerevisiae	DE42807	133.233	46.330	401.000	0.733	0.142	0.992
Saccharomyces cerevisiae	DE45866	132.969	46.445	398.000	0.729	0.144	0.993
Saccharomyces cerevisiae	465/2018	132.915	45.793	386.964	0.729	0.142	0.998
Saccharomyces cerevisiae	551/2018	139.615	51.146	406.000	0.735	0.145	0.994
Saccharomyces cerevisiae	2251/2018	125.976	39.852	358.000	0.744	0.139	0.995

Description of traits measured during high-throughput single-cell phenotyping

1	Cell size C101_A1B	51	Length from bud neck to the farthest point on mother cell C128_C	101	Relative distance of nuclear brightest point to cell center D148_A
2	Cell size C101_C	52	Fitness C13_A	102	Relative distance of nuclear brightest point to cell center D148_C
3	Contour length of cell C102_A1B	53	Fitness C13_A1B	103	Relative distance of nuclear gravity center in bud to bud center D149_C
4	Contour length of cell C102_C	54	Fitness C13_C	104	Relative distance of nuclear brightest point in bud to bud center D150_C
5	C103_A Long axis length of mother cell	55	Distance from nuclear center to mother tip D102_A	105	Distance ratio of two nuclei from neck D151_C
6	Long axis length of mother cell C103_A1B	56	Distance from nuclear center to mother tip D103_C	106	Mobility of nucleus in mother cell in nucleus D152_C
7	Long axis length of mother cell C103_C	57	Distance from nuclear center to mother tip D104_A1B	107	Mobility of nucleus in bud D153_C
8	Short axis length of mother cell C104_A	58	Ratio of D102 to C103 D105_A	108	Angle between C1D1−1 and C1C1−2 D154_A
9	Short axis length of mother cell C104_A1B	59	Ratio of D103 to C103 D106_C	109	Angle between C1D1−1 and C1C1−2 D154_A1B
10	Short axis length of mother cell C104_C	60	Ratio of D104 to C103 D107_A1B	110	Angle between C1D1−1 and C1C1−2 D154_C
11	Neck position C105_A1B	61	Distance from neck to mother cell's nucleus D108_C	111	Angle between C1D2−1 and C1C1−2 D155_A
12	Neck position C105_C	62	Distance from neck to bud's nucleus D109_C	112	Angle between C1D2−1 and C1C1−2 D155_A1B
13	Bud growth direction C106_A1B	63	Distance from neck to nucleus center D110_A1B	113	Angle between C1D2−1 and C1C1−2 D155_C
14	Bud growth direction C106_C	64	Ratio of D108 to C128 D112_C	114	Angle between C2D1−2 and C2C4−2 D156_C
15	Long axis length in bud C107_A1B	65	Ratio of D109 to C107 D113_C	115	Angle between C2D2−2 and C2C4−2 D157_C
16	Long axis length in bud C107_C	66	Ratio of D110 to C128 D114_A1B	116	Angle between D18−1D1−1 and D18−1C1−2 D158_C
17	Short axis length in bud C108_A1B	67	Distance between two nucleus D116_C	117	Angle between D19−1D2−1 and D19−1C1−2 D159_C
18	Short axis length in bud C108_C	68	Distance from mother cell's center to nucleus D117_A	118	Slope between two nuclei to neck position D162_C
19	Neck width C109_A1B	69	Distance from mother cell's center to mother cell's nucleus D117_C	119	Angle between D23D2−1 and D23C1 D163_C
20	Neck width C109_C	70	Distance from mother cell's center to nucleus center in A1B D118_A1B	120	Angle between D18−2D1−2 and D18−2C4−2 D166_C
21	Mother cell size C11−1_A	71	Distance from bud center to bud's nucleus D119_C	121	Angle between D19−2D2−2 and D19−2C4−2 D167_C
22	Mother cell size C11−1_A1B	72	Distance from bud nucleus to bud tip D121_C	122	Angle between M1D1−1 and M1C1 D169_A1B
23	Mother cell size C11−1_C	73	Ratio of D121 to C107 D123_C	123	Angle between M1D1−1 and M1C1 D169_C
24	Area of daughter cell C11−2_A1B	74	Distance between nuclear gravity center in mother and mother hip D125_C	124	Fitness to ellipse of the nucleus in the mother cell D17−1_A
25	Area of daughter cell C11−2_C	75	Distance between nuclear gravity center and mother hip D126_A1B	125	Fitness to ellipse of the nucleus in the mother cell D17−1_C
26	Length from bud tip to mother cell's long axis C110_A1B	76	Distance between nuclear brightest point and cell tip D127_A	126	Fitness to ellipse of the nucleus in the daughter cell D17−2_C
27	Length from bud tip to mother cell's long axis C110_C	77	Distance between nuclear brightest point in mother and mother tip D128_C	127	Fitness to ellipse of the nucleus D17−3_A1B
28	Length from bud tip to mother cell's short axis C111_A1B	78	Distance between nuclear brightest point and mother tip D129_A1B	128	Angle between M1D2−1 and M1C1 D170_A1B
29	Length from bud tip to mother cell's short axis C111_C	79	Distance between nuclear brightest point in mother and middle point of neck D130_C	129	Angle between M1D2−1 and M1C1 D170_C
30	Distance from neck to mother cell's center C112_A1B	80	Distance between nuclear brightest point in bud and middle point of neck D131_C	130	nucleus maximum radius in mother cell D173_A
31	Distance from neck to mother cell's center C112_C	81	Distance between nuclear brightest point and middle point of neck D132_A1B	131	nucleus maximum radius in mother cell D173_C
32	Distance from bud tip to mother cell's long axis along bud direction C113_A1B	82	Distance between two nuclear brightest points D134_C	132	nucleus maximum radius in bud D174_C
33	Distance from bud tip to mother cell's long axis along bud direction C113_C	83	Distance between nuclear brightest point and cell center D135_A	133	nucleus maximum radius D175_A1B
34	Roundness of bud C114_A1B	84	Distance between nuclear brightest point and cell center D135_C	134	nucleus diameter in mother cell D176_A
35	Roundness of bud C114_C	85	Distance between nuclear brightest point and mother center D136_A1B	135	nucleus diameter in mother cell D176_C
36	Roundness of mother cell C115_A	86	Distance between nuclear brightest point in bud and bud tip D137_C	136	nucleus diameter in bud D177_C
37	Roundness of mother cell C115_A1B	87	Distance between nuclear brightest point in bud and bud tip D139_C	137	nucleus diameter D178_A1B
38	Roundness of mother cell C115_C	88	Area of nucleus region in mother cell D14−1_A	138	nucleus minimum radius in mother cell D179_A
39	Ratio of roundness of mother cell to that of bud C116_A1B	89	Area of nucleus region in mother cell D14−1_C	139	nucleus minimum radius in mother cell D179_C
40	Ratio of roundness of mother cell to that of bud C116_C	90	Area of nucleus region in bud D14−2_C	140	nucleus minimum radius in bud D180_C
41	Ratio of the countour length C117_A1B	91	Area of nucleus region D14−3_A1B	141	nucleus minimum radius D181_A1B
42	Ratio of the countour length C117_C	92	Area of nucleus region D14−3_C	142	nucleus roundness in mother cell D182_A
43	Ratio of the cell sizes C118_A1B	93	Distance between nuclear brightest point in mother and mother hip D141_C	143	nucleus roundness in mother cell D182_C
44	Ratio of the cell sizes C118_C	94	Distance between nuclear brightest point and mother hip D142_A1B	144	nucleus roundness in bud D183_C
45	Contour length of mother cell C12−1_A	95	Nucleus border point close to neck D143_C	145	nucleus roundness D184_A1B
46	Contour length of mother cell C12−1_A1B	96	Nucleus border point close to neck on bud's nucleus D144_C	146	total length of segment connecting the respective point on the outline of the mother cell intersected by the line connecting the mother cell nucleus center with the midpoint of the neck and the segment connecting the midpoint of the neck to the analogous point in the bud D185_C
47	Contour length of mother cell C12−1_C	97	Distance between nuclear outline point C7 and mother hip D145_C	147	Relative distance of two nuclear brightest points to middle point of neck D186_C
48	Contour length of daughter cell C12−2_A1B	98	Distance between nuclear outline point C8 in bud and bud tip D146_C	148	Distance between nuclear gravity center and brightest point D190_A1B
49	Contour length of daughter cell C12−2_C	99	Relative distance of nuclear gravity center to cell center D147_A	149	nuclei size ratio D197_C
50	Length from bud neck to the farthest point on mother cell C128_A1B	100	Relative distance of nuclear gravity center to cell center D147_C		

Traits important for single-cell phenotyping PCA. List of traits with the highest absolute value PC loadings for each of the six main PCs obtained for single-cell phenotyping.

PC1		PC2		PC3		PC4		PC5		PC6	
Trait	PC loading	Trait	PC loading	Trait	PC loading	Trait	PC loading	Trait	PC loading	Trait	PC loading
D186_C	-0.136	D14-1_A	-0.16493	D141_C	-0.20434	D113_C	-0.19212	C107_A1B	0.222062	D182_C	0.307633
D185_C	-0.1356	D179_A	-0.16321	D125_C	-0.19926	C114_A1B	-0.18617	C116_A1B	0.216002	D17-1_C	0.292928
C12-1_A	-0.13307	D180_C	-0.1627	D128_C	-0.19872	C108_C	0.184973	C12-2_A1B	0.211158	D183_C	0.21688
C11-1_A1B	-0.13281	D14-3_C	-0.16202	D156_C	-0.19812	C13_C	-0.18405	C11-2_A1B	0.209006	D150_C	0.216855
C102_C	-0.13218	D14-3_A1B	-0.162	D103_C	-0.19442	C109_A1B	0.183141	C117_A1B	0.192861	D149_C	0.20991
C11-1_A	-0.13197	D14-2_C	-0.16113	D152_C	0.178876	D154_C	0.181454	D17-1_A	-0.19147	D197_C	-0.18847
D109_C	-0.13139	D181_A1B	-0.16001	D112_C	0.177224	C108_A1B	0.175769	C118_A1B	0.190854	D17-2_C	0.182781
C12-1_A1B	-0.13135	D179_C	-0.15947	D106_C	-0.17606	D154_A	-0.17572	D114_A1B	0.177103	D135_A	0.167241
C103_A	-0.13109	D173_A	-0.15941	D151_C	-0.17519	D123_C	0.17404	C114_A1B	0.161997	D119_C	0.154448
D116_C	-0.13099	D176_A	-0.15869	D145_C	-0.16736	C113_C	0.159034	C110_A1B	0.148458	D154_A	-0.15314
D134_C	-0.13085	D178_A1B	-0.1579	D162_C	-0.15905	C118_C	0.159018	D154_A1B	0.144525	C109_A1B	-0.14369
C101_C	-0.13074	D107_A1B	0.157187	D163_C	-0.15865	C13_A1B	-0.15452	D17-2_C	-0.14407	D137_C	0.137245
D144_C	-0.13002	D14-1_C	-0.15688	D167_C	-0.15712	C117_C	0.149354	D167_C	0.137712	C13_A	0.132817
D131_C	-0.12986	D175_A1B	-0.15666	D157_C	-0.15503	D139_C	0.147335	C13_A1B	0.136971	D155_A	-0.12721
C111_C	-0.12938	D174_C	-0.1557	D147_C	0.146532	C13_A	-0.14676	D182_A	-0.13392	D113_C	-0.12533

Yeast group comparisons for single-cell phenotyping. List of traits obtained for single-cell phenotyping and uncorrected p-values for group level (commercial, mycosis, non-mycosis) comparisons with Kruskal-Wallis test.

Trait	p-value	Trait	p-value	Trait	p-value	Trait	p-value	Trait	p-value
D149_C	0.045498	D135_A	0.220807	D112_C	0.312746	D144_C	0.385879	C113_A1B	0.582849
D150_C	0.047989	C13_C	0.221966	D134_C	0.312746	C110_A1B	0.390663	D126_A1B	0.617659
D182_C	0.057558	C102_C	0.238791	D135_C	0.312746	D159_C	0.39259	D176_A	0.623129
C104_C	0.064164	D132_A1B	0.241288	D182_A	0.312746	C101_A1B	0.393556	D142_A1B	0.635545
C104_A1B	0.077048	D180_C	0.245079	C106_C	0.315132	D156_C	0.394525	C13_A	0.652325
D137_C	0.086275	D146_C	0.245716	C13_A1B	0.320762	C114_A1B	0.397443	D173_A	0.662227
C11-1_A1B	0.089416	D148_C	0.251517	D127_A	0.324838	D153_C	0.397443	D152_C	0.663649
D119_C	0.090903	C108_C	0.252169	C117_C	0.326481	C115_A	0.406307	D184_A1B	0.665072
C104_A	0.1098	D17-1_C	0.252169	C118_C	0.326481	D17-2_C	0.406307	D179_C	0.68227
D121_C	0.11222	D174_C	0.252169	D170_A1B	0.326481	C128_A1B	0.410299	D155_A1B	0.695312
D155_C	0.13135	D177_C	0.252169	D116_C	0.327305	C117_A1B	0.411302	D14-3_A1B	0.748511
D197_C	0.133138	D14-2_C	0.254794	D117_C	0.328131	C106_A1B	0.413314	D179_A	0.769536
D17-3_A1B	0.154794	D163_C	0.254794	C109_A1B	0.33734	D169_A1B	0.42453	D14-1_A	0.780092
D17-1_A	0.156044	C11-1_A	0.258109	D105_A	0.33989	D154_A	0.425562	D169_C	0.81637
D185_C	0.158147	C113_C	0.264176	D117_A	0.33989	D167_C	0.431797	C102_A1B	0.834467
D186_C	0.158147	D139_C	0.266227	D162_C	0.33989	D176_C	0.431797	D181_A1B	0.834467
D154_C	0.161137	C111_C	0.273871	C110_C	0.3416	D158_C	0.440227	D175_A1B	0.838976
D123_C	0.16418	D128_C	0.278838	C116_C	0.347643	C118_A1B	0.442356	D170_C	0.850961
D113_C	0.16506	C11-2_A1B	0.280992	D145_C	0.347643	D155_A	0.442356	D14-1_C	0.858414
D151_C	0.16506	C12-1_A1B	0.28316	D190_A1B	0.351137	C128_C	0.445564	D178_A1B	0.858414
C12-2_C	0.169976	D110_A1B	0.28316	D147_A	0.352896	D14-3_C	0.447713	C12-1_C	0.861385
C107_C	0.172715	D125_C	0.288278	C105_C	0.353778	C112_A1B	0.452037	D118_A1B	0.874673
C109_C	0.1741	D141_C	0.288278	D147_C	0.359112	D102_A	0.452037	D136_A1B	0.920262
C11-2_C	0.198769	D148_A	0.289756	C108_A1B	0.36361	D173_C	0.46967	D130_C	0.932391
D109_C	0.198769	D166_C	0.289756	D103_C	0.36361	C103_C	0.486692	D104_A1B	0.933714
D131_C	0.198769	C101_C	0.295734	C12-2_A1B	0.369071	C112_C	0.486692	D108_C	0.933714
C12-1_A	0.200878	D114_A1B	0.30029	C105_A1B	0.377395	C11-1_C	0.519743	D129_A1B	0.933714
C103_A	0.205697	C115_C	0.304908	C107_A1B	0.377395	C103_A1B	0.522167	D107_A1B	0.936343
D183_C	0.21229	C115_A1B	0.311164	C111_A1B	0.377395	D154_A1B	0.536889	D143_C	0.96692
C116_A1B	0.216226	D106_C	0.312746	C114_C	0.377395	D157_C	0.555715		

Phenotypic clustering of isolates based on FlowSight results (mean area, 99.9% quantile value for area, mean aspect ratio). Unit variance scaling is applied to rows. Rows are clustered using correlation distance and average linkage. Columns are clustered using Euclidean distance and Ward linkage.

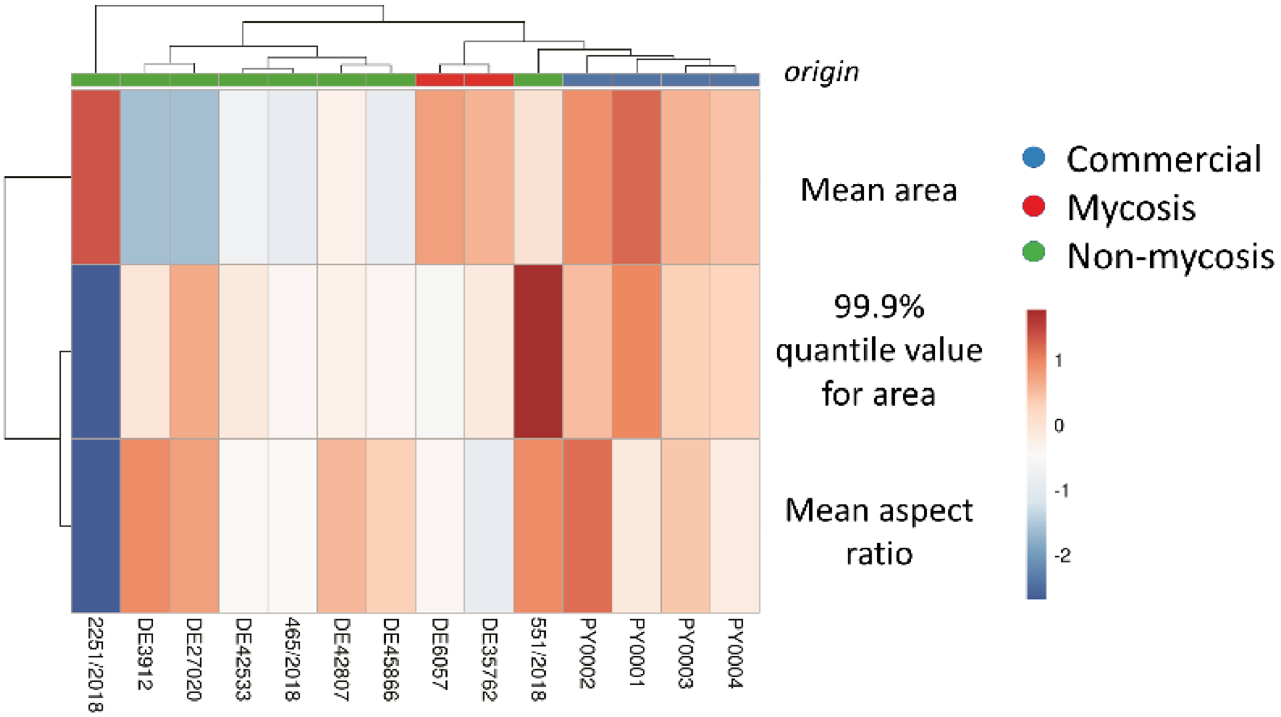

Plot showing the area distribution of events recorded by FlowSight analysis. Plots are colored according to isolate group.

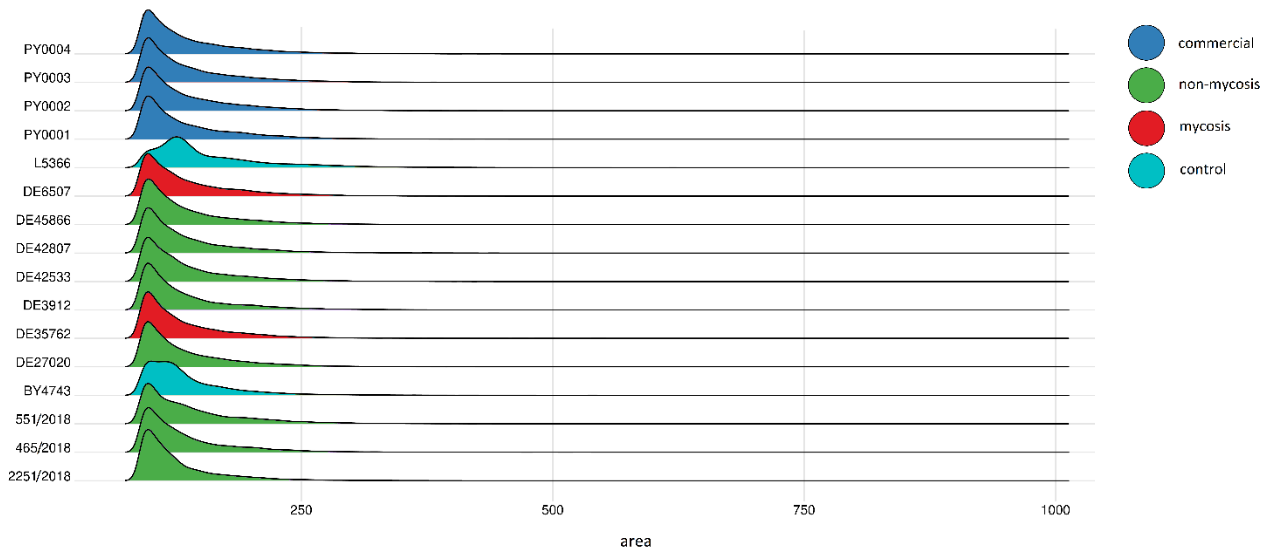

Single cell high-throughput phenotyping: ratio of mother cell long and short axis (C115_*A*). The means of measurements (bars) and individual measurements (dots) are shown for each isolate. Isolates are colored according to isolate group.

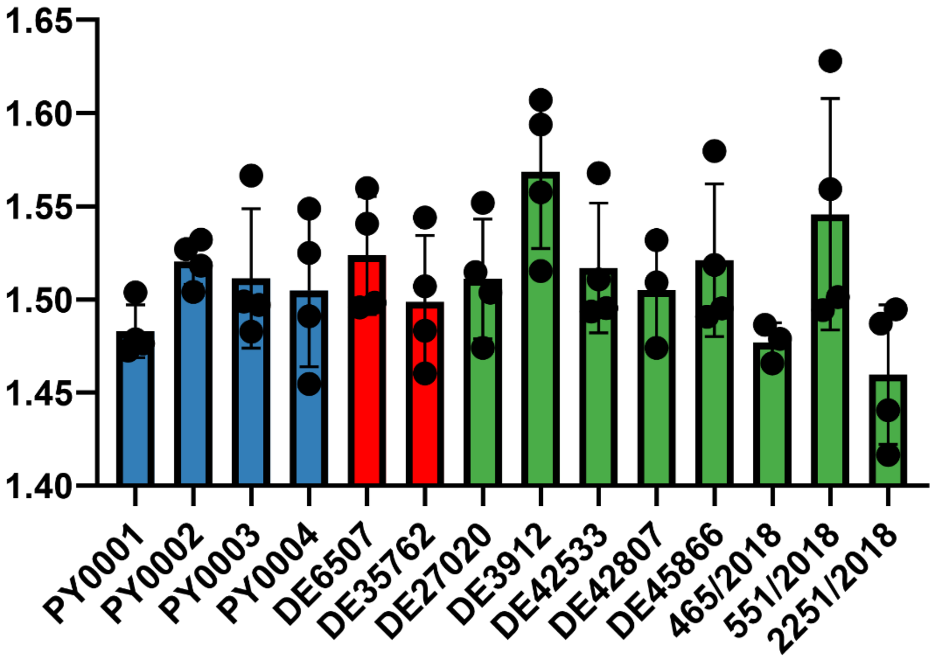

Supplementary File S4. Interactions with epithelial model. For MTT assay, fluorescent signal relative to uninfected control is given in percentage (18 wells/experiment, mean values)**.**

Isolate	Adhesion		Damage		Transmission														Biofilm in upper compartment, 2-6 days
	Adherent cell number/sq. mm, washed 2 h after infection (3 replicates, 9 viewfields)		MTT assay mean correc-ted values (%)	MTT assay S.D.	CFU/ml in lower compartment (mean of 3 replicates)														
	Cell number	SD			0 h	S.D.	24 h	S.D.	48 h	S.D.	72 h	S.D.	96 h	S.D.	120 h	S.D.	144 h	S.D.	
SC5314	109.63	21.58	0.48	1.180	0	0	0	0	1.06866×10^5	2.3044×10^4	5.37466×10^5	1.04514×10^5	8.933333×10^6	1.1351798×10^7	6.033333×10^6	$3.695041*10^6$	$2.5*10^6$	1.946792×10^6	yes
PY0001	0	0	96.72	10.090	0	0	0	0	0	0	0	0	0	0	0	0	0	0	no
PY0002	0	0	87.35	11.094	0	0	0	0	0	0	0	0	0	0	0	0	0	0	no
PY0003	0	0	102.21	13.405	0	0	0	0	0	0	0	0	0	0	0	0	0	0	no
PY0004	0	0	103.83	16.224	0	0	0	0	0	0	0	0	0	0	0	0	0	0	no
DE6057	0	0	85.47	17.564	0	0	0	0	0	0	0	0	0	0	0	0	0	0	no
DE35762	0	0	95.86	9.662	0	0	0	0	0	0	0	0	0	0	0	0	0	0	no
DE27020	0	0	86.41	17.564	0	0	0	0	0	0	0	0	0	0	0	0	0	0	no
DE3912	0	0	92.64	8.046	0	0	0	0	0	0	0	0	170	147.99	626.67	506.49	1600	2109.41	no
DE42533	0	0	83.26	12.360	0	0	0	0	0	0	0	0	0	0	0	0	0	0	no
DE42807	0	0	96.99	18.686	0	0	0	0	0	0	0	0	0	0	0	0	0	0	no
DE45866	0	0	86.37	6.7917	0	0	0	0	0	0	0	0	0	0	0	0	0	0	no
465/2018	0	0	103.12	10.943	0	0	0	0	0	0	0	0	0	0	0	0	0	0	no
551/2018	0	0	104.02	14.303	0	0	0	0	0	0	0	0	0	0	0	0	12766.67	10767.70	no
2251/2018	0	0	90.85	21.547	0	0	0	0	0	0	0	0	0	0	0	0	0	0	no

Respresentative microphotographs of epithelial model and adhered yeasts. For each image pair, left side image shows monolayer with phase contrast microscopy, right side image shows the same viewfield with fluorescent microscopy. Blue color marks the stained nuclei on the fluorescent images. Scale bar represent 100 µm.

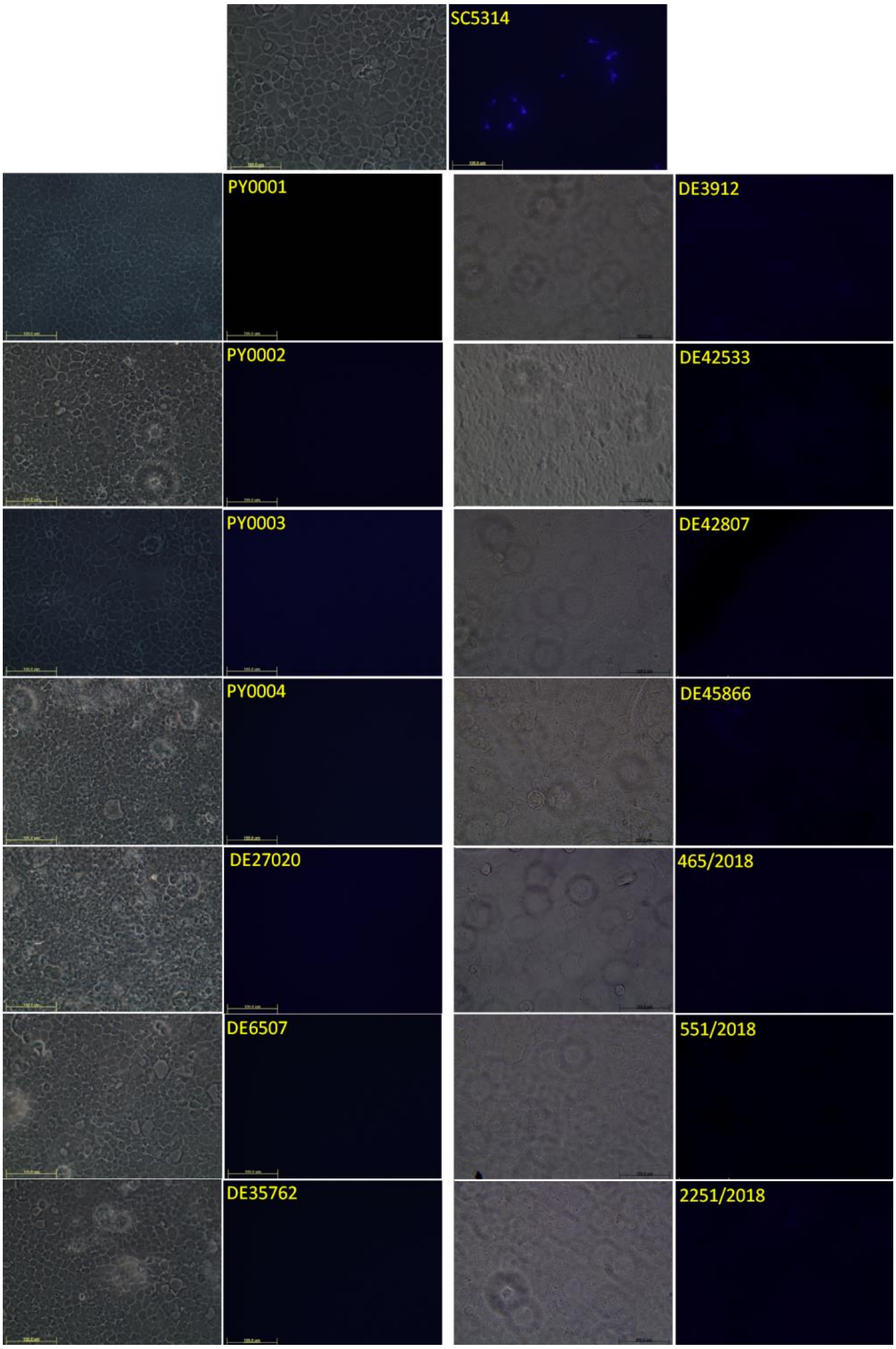

Transmigration through Caco-2 monolayer (Transwell assay). CFU numbers (with standard deviation) are shown in the lower compartment. As a positive control, the type strain of *C. albicans*, SC5314 was used.

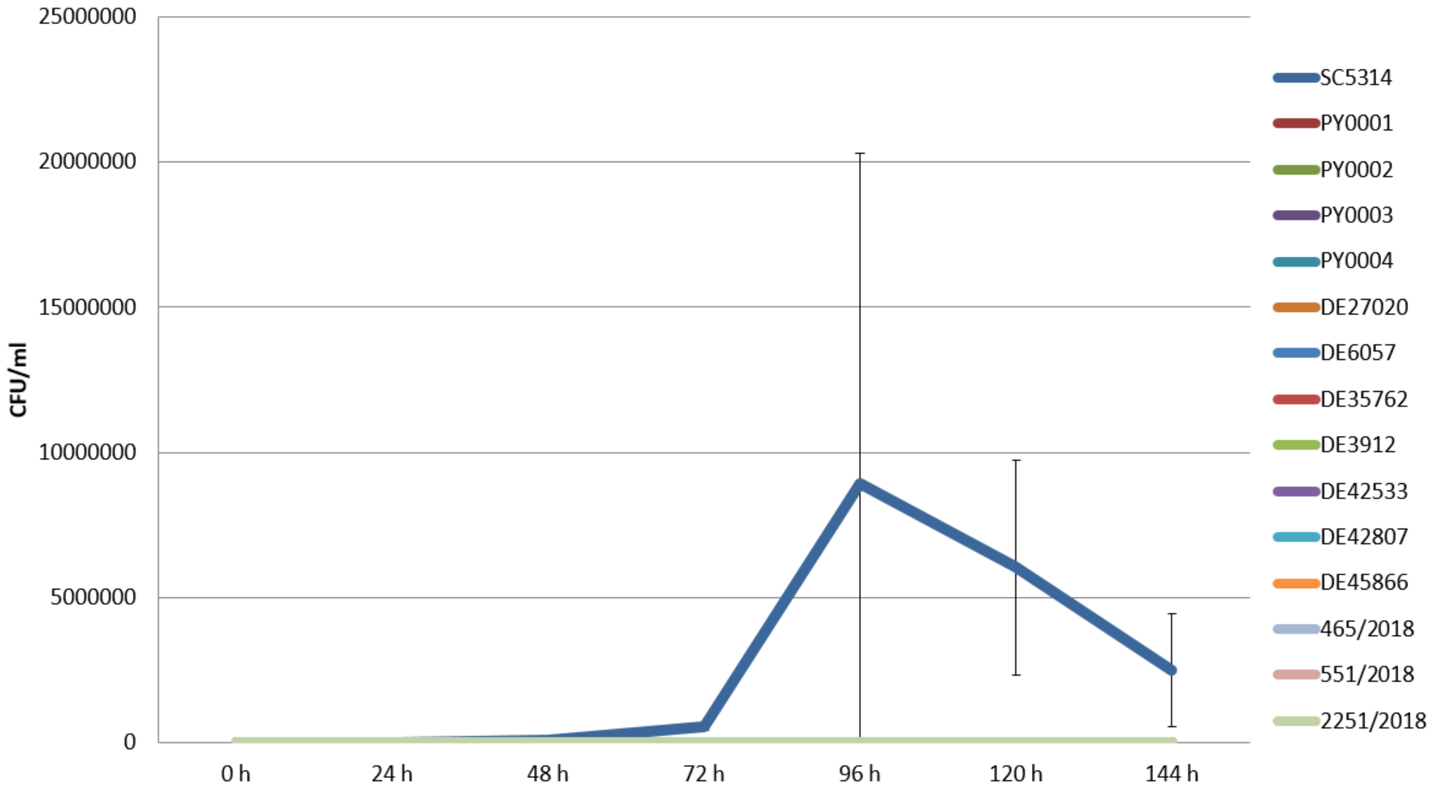

Supplementary File S5. Interactions with primary dendritic cells: phagocytic activity.

Sample		Phagocytic activity of DCs (fluorescence) corrected with uninfected and 4°C controls	
Species	Strain/Isolate	mean from 4 donors	S.D.
Candida albicans	SC5314	46.70	11.00
Saccharomyces cerevisiae	PY0001	42.77	1.61
Saccharomyces cerevisiae	PY0002	47.97	4.09
Saccharomyces cerevisiae	PY0003	42.88	4.33
Saccharomyces cerevisiae	PY0004	49.31	4.48
Saccharomyces cerevisiae	DE27020	41.79	3.64
Saccharomyces cerevisiae	DE6057	46.08	6.38
Saccharomyces cerevisiae	DE35762	47.56	7.73

Donor-dependent differences in phagocytic activity. Phagocytic activity of DCs (fluorescence) corrected with uninfected and 4°C controls shown for each donor.

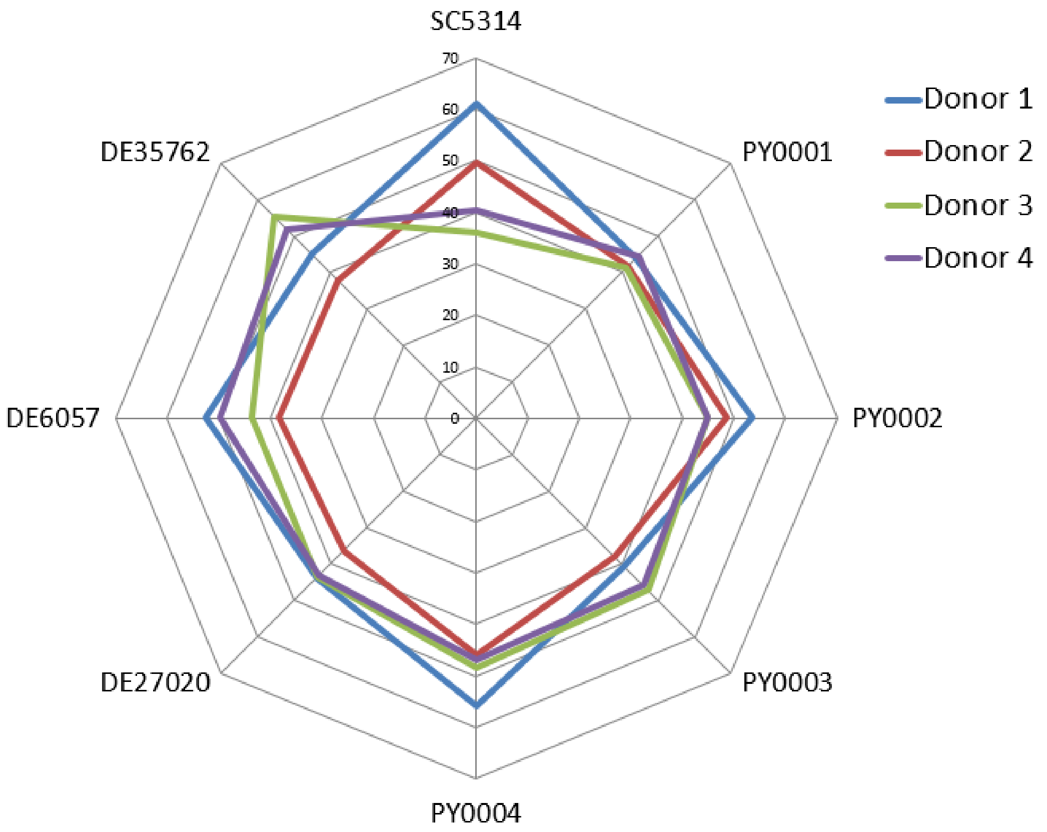

Supplementary File S6. Interactions with primary dendritic cells: DC activation

Sample		Phenotype: fold change in fluorescence (CD40, CD80, CD86, HLA-DQ), or change in percentage of positive cells (CD83)									
		CD40 (costimulatory)		CD80 (costimulatory)		CD86 (costimulatory)		CD83 (maturation marker)		HLA-DQ (antigen-presenting)	
Species	Strain/Isolate	mean from 4 donors	S.D.	mean from 4 donors	S.D.	mean from 4 donors	S.D.	mean from 4 donors	S.D.	mean from 4 donors	S.D.
Candida albicans	SC5314	2.19	0.41	2.85	1.01	38.95	30.09	1.87	0.52	2.75	1.69
Saccharomyces cerevisiae	PY0001	2.27	0.63	2.67	0.89	40.52	32.70	1.88	0.47	4.02	1.70
Saccharomyces cerevisiae	PY0002	2.27	0.85	2.93	1.02	48.94	39.35	1.96	0.54	4.33	2.20
Saccharomyces cerevisiae	PY0003	2.28	0.94	2.70	0.86	41.37	37.42	1.92	0.55	4.11	1.64
Saccharomyces cerevisiae	PY0004	2.36	0.69	2.68	0.91	41.42	32.68	1.87	0.53	3.19	0.99
Saccharomyces cerevisiae	DE27020	2.10	0.93	2.64	0.80	39.45	29.95	1.81	0.52	3.42	0.93
Saccharomyces cerevisiae	DE6057	2.33	0.62	2.76	1.00	44.28	36.30	2.00	0.62	3.18	0.92
Saccharomyces cerevisiae	DE35762	2.37	1.14	2.73	0.89	42.54	35.62	1.98	0.62	5.47	1.67

Sample		Cytokine/chemokine production: change in concentration (pg/mL)									
		IL-6 (proinflammatory)		IL-8 (chemokine)		TNFα (proinflammatory)		IL-12 (proinflammatory)		IL-1β (proinflammatory)	
Species	Strain/Isolate	mean from 4 donors	S.D.	mean from 4 donors	S.D.	mean from 4 donors	S.D.	mean from 2 donors	S.D.	mean from 3 donors	S.D.
Candida albicans	SC5314	30581.25	8940.39	29007.94	5701.79	28930.56	3065.57	6546.67	659.97	564.56	378.99
Saccharomyces cerevisiae	PY0001	20675.00	7417.43	20861.11	10894.75	22513.89	9568.00	4380.00	424.26	401.40	368.17
Saccharomyces cerevisiae	PY0002	25893.75	9101.70	23503.97	9234.78	27986.11	8887.35	4113.33	471.40	447.02	289.40
Saccharomyces cerevisiae	PY0003	21456.25	6110.22	19920.63	9473.68	23069.44	10060.36	3780.00	329.98	371.58	353.51
Saccharomyces cerevisiae	PY0004	29956.25	9031.39	24174.60	5005.94	29458.33	3540.91	5446.67	329.98	361.05	256.51
Saccharomyces cerevisiae	DE27020	21893.75	6868.51	22087.30	7912.62	22625.00	5970.24	3913.33	518.54	354.04	332.47
Saccharomyces cerevisiae	DE6057	23518.75	8550.42	23726.19	8321.48	26611.11	5877.47	4646.67	565.69	376.84	142.12
Saccharomyces cerevisiae	DE35762	26456.25	8874.79	23718.25	8757.21	29013.89	8765.80	5180.00	1084.23	438.25	101.05

Donor dependent differences in phenotype of DCs. Fold changes in fluorescence for CD40, CD80, CD86, and HLA-DQ markers shown for each donor and each isolate. For CD83, fold change in percentage of positive cells is shown for each donor and each isolate.

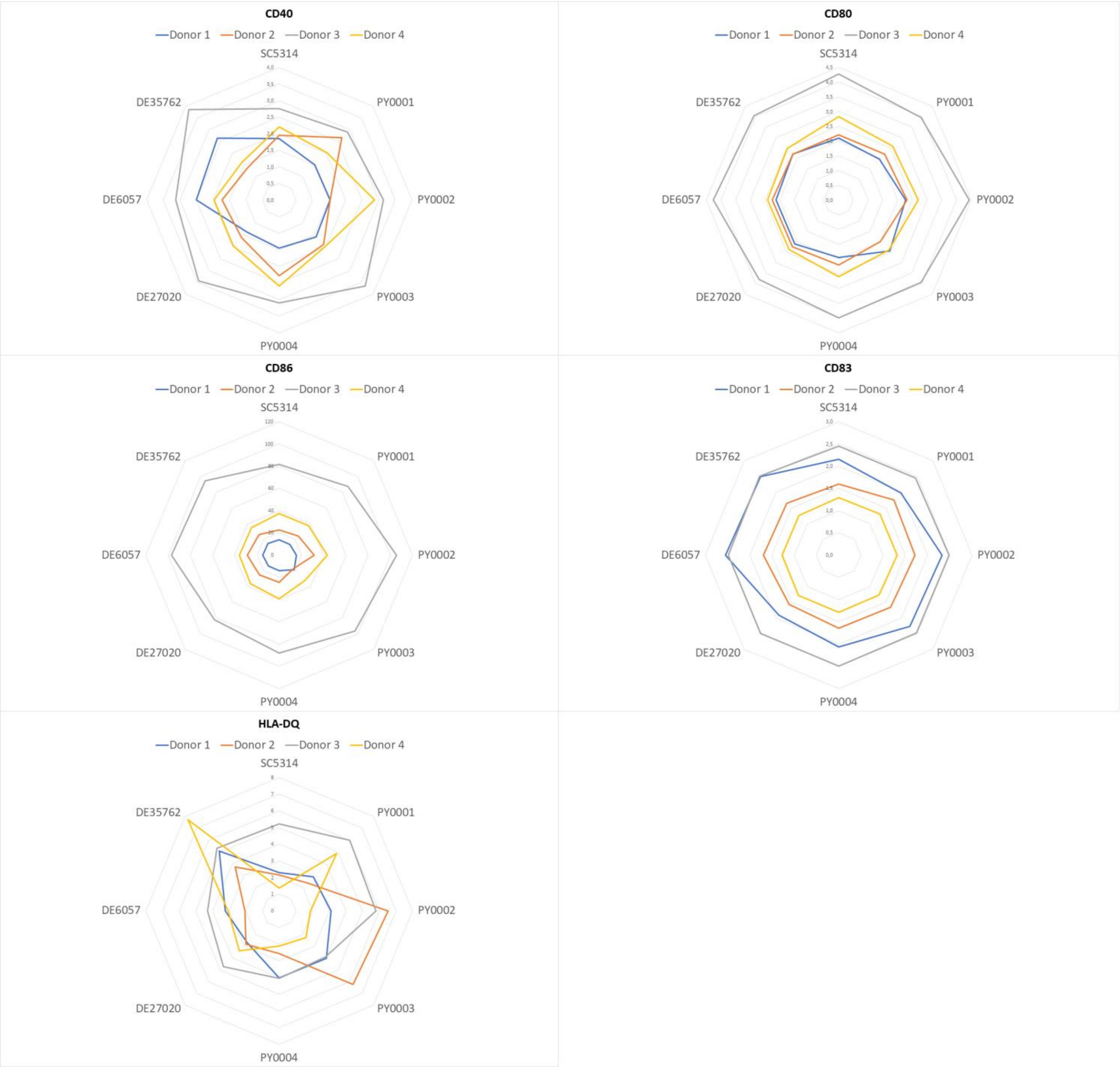

Donor dependent differences in cytokine/chemokine production. Changes in production (pg/mL) for each measured molecule shown for each donor.

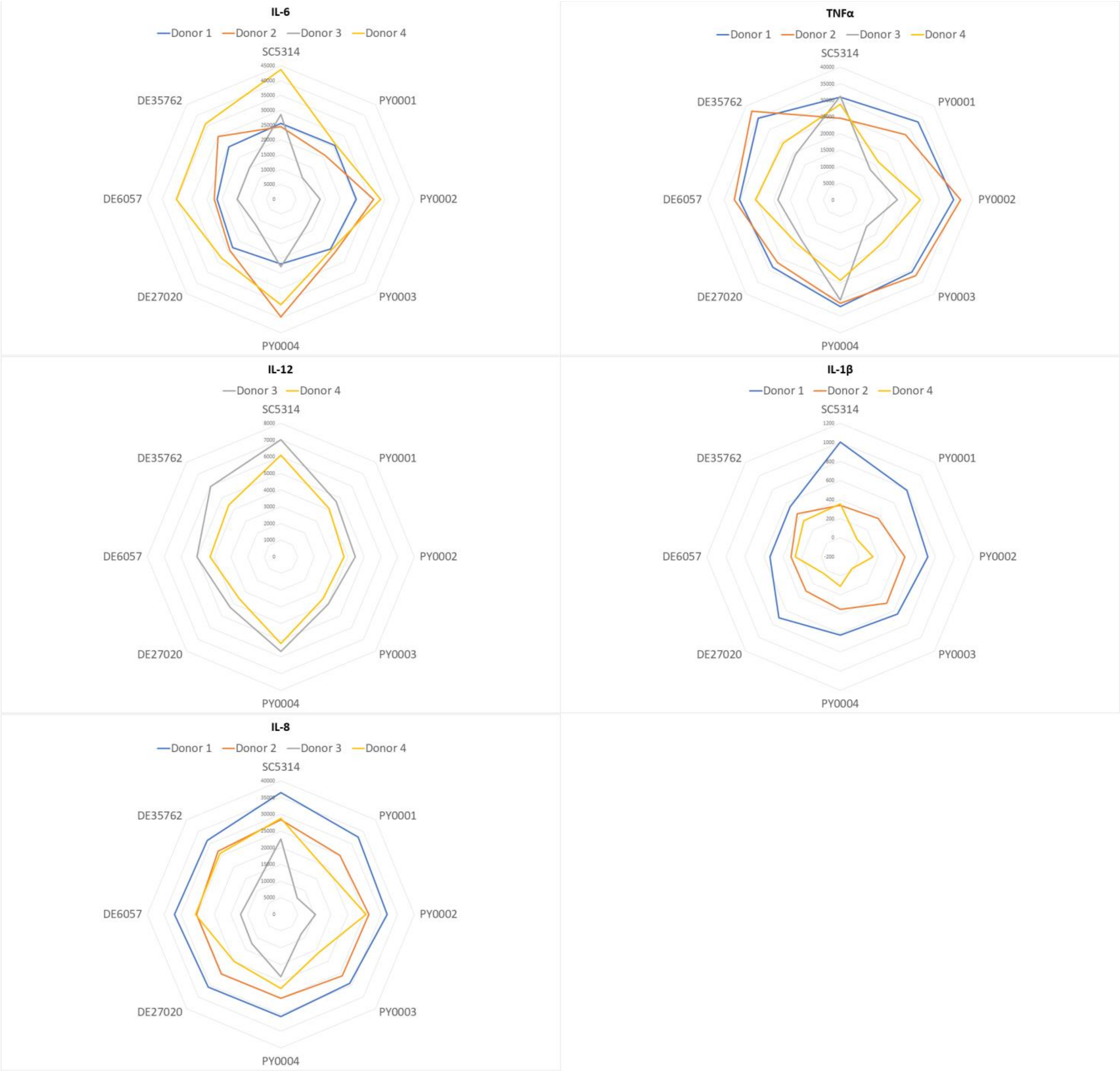

Supplementary File S7. Primary T-cell activation by activated moDCs.

Sample		Number of cytokine producing activated T-cells exposed by activated DCs (spot number)			
		T- cells positive for IL-17 cytokine		T-cells positive for IFN-γ cytokine	
Species	Cell/Strain/Isolate	Mean from 3 donors	S.D.	Mean from 2 donors	S.D.
n/a	T-cell control	5.89	6.95	0.33	0.82
n/a	DC control	47.11	31.01	0.17	0.41
Candida albicans	SC5314	252.78	40.43	98.33	79.69
Saccharomyces cerevisiae	PY0001	82.56	53.24	1.33	1.21
Saccharomyces cerevisiae	PY0002	90.78	44.61	1.17	1.94
Saccharomyces cerevisiae	PY0003	103.89	80.09	2.00	0.89
Saccharomyces cerevisiae	PY0004	82.00	50.19	2.33	1.63
Saccharomyces cerevisiae	DE27020	90.67	42.91	2.50	2.17
Saccharomyces cerevisiae	DE6057	117.78	49.56	1.83	1.72
Saccharomyces cerevisiae	DE35762	130.67	61.60	4.00	2.37

Primary T-cell activation by activated moDCs: ELISPOT images.

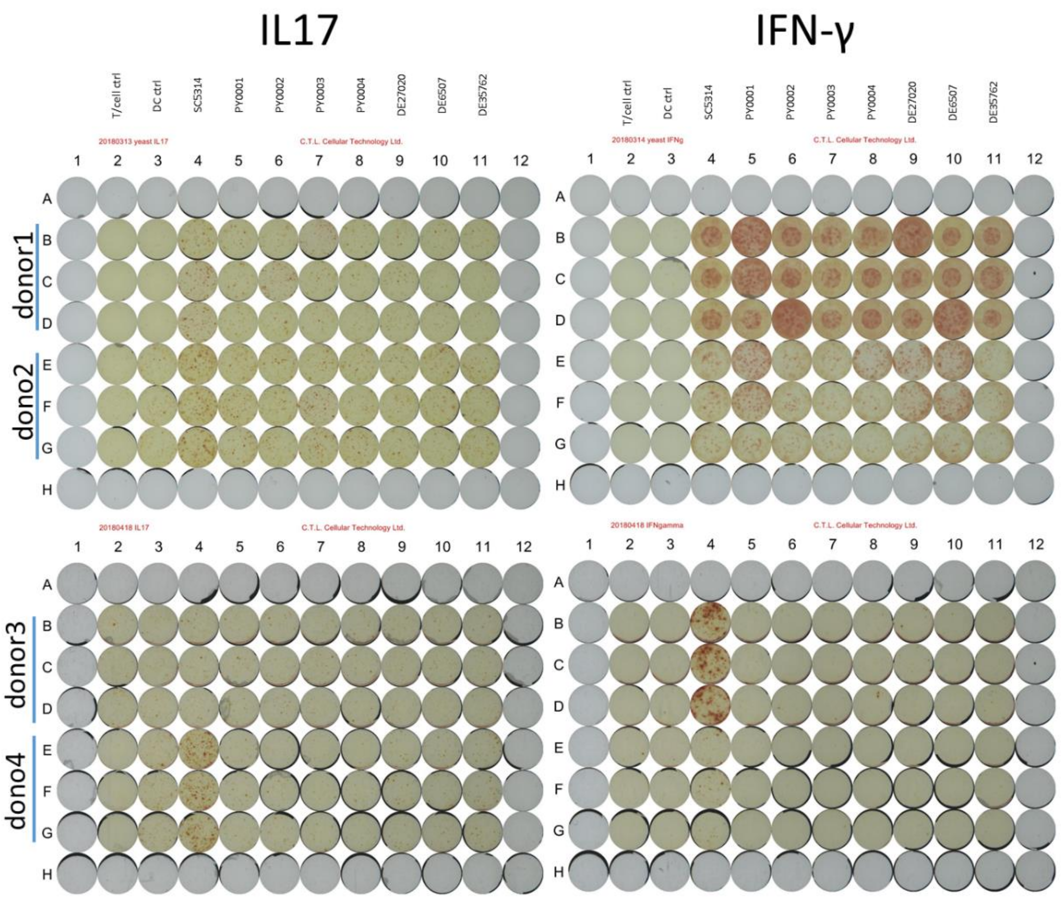

Phenotypic clustering of isolates based on immunological interactions. a. With *C. albicans* SC5314 used as control. b. clustering and heatmap only generated for *S. 'boulardii'*. Unit variance scaling is applied to rows. Rows are clustered using correlation distance and average linkage. Columns are clustered using Euclidean distance and average linkage. Donor means were used for clustering.

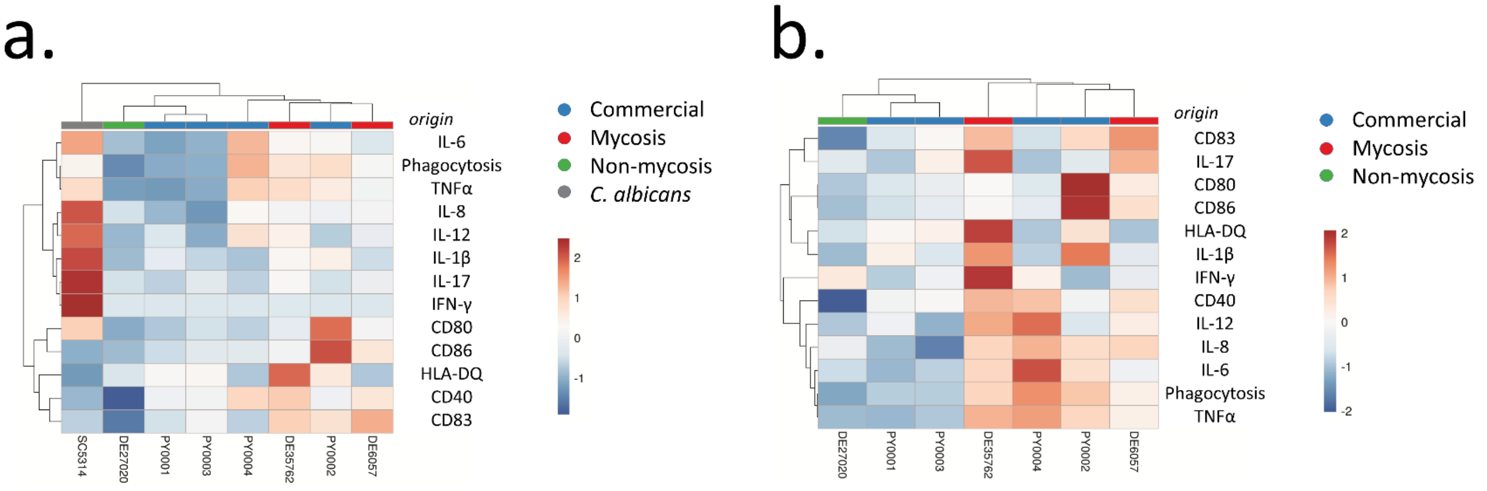

Principal component analysis of isolates based on immunological interactions. Unit variance scaling is applied to various measured DC and T-cell characteristics. SVD (Singular Value Decomposition) with imputation is used to calculate principal components. Prediction ellipses are such that with probability 0.95, a new observation from the same group will fall inside the ellipse.

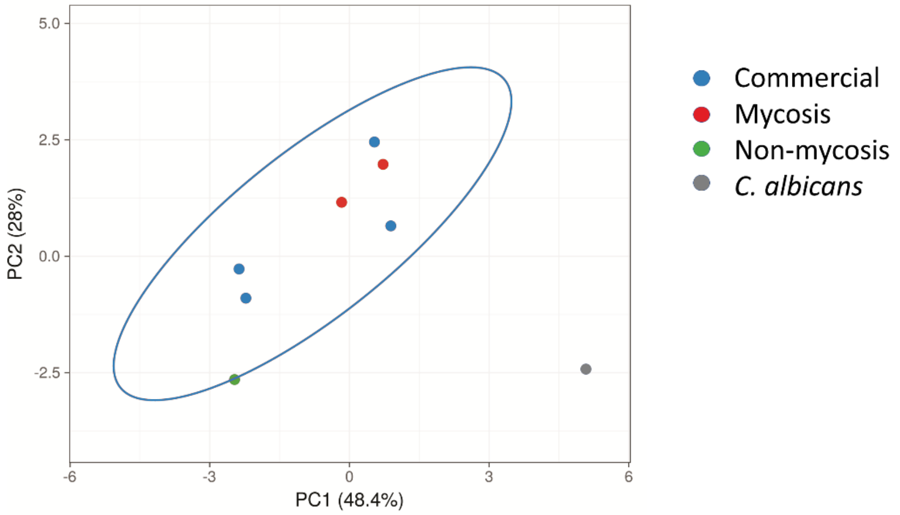

Supplementary file S8. *Galleria* larva pathogenicity model

Sample		Number of larvae surviving					Statistical comparison of survival: p value of Goodness of Fit, using χ^2 distribution (righ-tailed) and log rank test p values (in brackets) for differences in survival curves of isolates																
**Species**	**Strain/Isolate**	**0 h**	**24 h**	**48 h**	**72 h**	**96 h**	**Strain/Isolate**	PBS	SC5314	PY0001	PY0002	PY0003	PY0004	DE27020	DE6057	DE35762	DE3912	DE42807	DE42533	DE45866	465/2018	551/2018	2251/2018
PBS control		40	36	35	35	35	PBS	-	-	-	-	-	-	-	-	-	-	-	-	-	-	-	-
Candida albicans	SC5314	40	2	1	1	1	SC5314	6.66655e-8<br>(3.66374e-15)	-	-	-	-	-	-	-	-	-	-	-	-	-	-	-
Saccharomyces cerevisiae	PY0001	40	28	27	24	24	PY0001	0.0110665<br>(0.00574632)	0.000196669<br>(6.55143e-10)	-	-	-	-	-	-	-	-	-	-	-	-	-	-
Saccharomyces cerevisiae	PY0002	40	28	26	26	25	PY0002	0.0185868<br>(0.0102504)	0.000174164<br>(6.32693e-10)	0.859804<br>(0.840200)	-	-	-	-	-	-	-	-	-	-	-	-	-
Saccharomyces cerevisiae	PY0003	40	32	26	23	22	PY0003	0.00333433<br>(0.00188633)	0.0000330045<br>(3.15382e-11)	0.774846<br>(0.752065)	0.652176<br>(0.618567)	-	-	-	-	-	-	-	-	-	-	-	-
Saccharomyces cerevisiae	PY0004	40	27	27	27	26	PY0004	0.0333922<br>(0.0185950)	0.000231339<br>(8.92706e-10)	0.709469<br>(0.665563)	0.852484<br>(0.828341)	0.512121<br>(0.466721)	-	-	-	-	-	-	-	-	-	-	-
Saccharomyces cerevisiae	DE27020	40	33	32	32	32	DE27020	0.391894<br>(0.364216)	0.00000172181<br>(3.77032e-13)	0.0856848<br>(0.0566066)	0.125369<br>(0.0878569)	0.0379165<br>(0.0257626)	0.187290<br>(0.135579)	-	-	-	-	-	-	-	-	-	-
Saccharomyces cerevisiae	DE6057	40	31	30	28	25	DE6057	0.0474656<br>(0.0380626)	0.00000573223<br>(1.30274e-12)	0.466321<br>(0.424433)	0.604660<br>(0.570140)	0.268762<br>(0.236224)	0.775740<br>(0.751840)	0.266044<br>(0.236436)	-	-	-	-	-	-	-	-	-
Saccharomyces cerevisiae	DE35762	40	35	31	28	26	DE35762	0.0296850<br>(0.0240913)	0.00000193699<br>(2.19158e-13)	0.568261<br>(0.536842)	0.704513<br>(0.681679)	0.351323<br>(0.318745)	0.883129<br>(0.872928)	0.198292<br>(0.176034)	0.831635<br>(0.822843)	-	-	-	-	-	-	-	-
Saccharomyces cerevisiae	DE3912	40	40	37	37	37	DE3912	0.425554<br>(0.418456)	7.29431e-10<br>(5.55112e-16)	0.000973098<br>(0.000437926)	0.00187550<br>(0.000992675)	0.000164814<br>(0.0000828110)	0.00437667<br>(0.00227938)	0.101376<br>(0.0913119)	0.00602867<br>(0.00463382)	0.00276236<br>(0.00208457)	-	-	-	-	-	-	-
Saccharomyces cerevisiae	DE42807	40	39	38	37	34	DE42807	0.810396<br>(0.807205)	6.69058e-9<br>(2.22045e-16)	0.0127884<br>(0.00822102)	0.0232283<br>(0.0158562)	0.00319396<br>(0.00202786)	0.0444841<br>(0.0311714)	0.508968<br>(0.495635)	0.0643450<br>(0.0551621)	0.0357506<br>(0.0303420)	0.299041<br>(0.292534)	-	-	-	-	-	-
Saccharomyces cerevisiae	DE42533	40	37	37	37	36	DE42533	0.728469<br>(0.720410)	1.86631e-8<br>(6.66134e-16)	0.00411607<br>(0.00193991)	0.00761530<br>(0.00384908)	0.000982120<br>(0.000529038)	0.0150553<br>(0.00751334)	0.233189<br>(0.209998)	0.0195569<br>(0.0148473)	0.0108974<br>(0.00858152)	0.178254<br>(0.669357)	0.535309<br>(0.528702)	-	-	-	-	-
Saccharomyces cerevisiae	DE45866	40	40	38	37	37	DE45866	0.436745<br>(0.429833)	8.07003e-10<br>(4.44089e-16)	0.00109176<br>(0.000538944)	0.00203149<br>(0.00107344)	0.000218557<br>(0.000110074)	0.00446271<br>(0.00238281)	0.105002<br>(0.0948863)	0.00655835<br>(0.00524195)	0.00331990<br>(0.00254711)	0.966596<br>(0.966078)	0.311219<br>(0.306165)	0.679979<br>(0.677704)	-	-	-	-
Saccharomyces cerevisiae	465/2018	40	36	34	33	33	465/2018	0.560880<br>(0.547326)	1.36922e-7<br>(6.55032e-15)	0.0401810<br>(0.0262224)	0.0618990<br>(0.0428718)	0.0147186<br>(0.00966505)	0.101588<br>(0.0726638)	0.764392<br>(0.752183)	0.154645<br>(0.136994)	0.104724<br>(0.0916303)	0.160899<br>(0.153383)	0.697142<br>(0.691945)	0.345770<br>(0.333073)	0.171522<br>(0.164021)	-	-	-
Saccharomyces cerevisiae	551/2018	40	38	38	38	38	551/2018	0.238678<br>(0.225461)	3.21136e-9<br>(8.88178e-16)	0.000545156<br>(0.000196175)	0.00106301<br>(0.000411744)	0.000103614<br>(0.0000472886)	0.00234639<br>(0.000852355)	0.0548978<br>(0.0439953)	0.00273440<br>(0.00187843)	0.00139028<br>(0.00101403)	0.679708<br>(0.676515)	0.154512<br>(0.150431)	0.411110<br>(0.400986)	0.671346<br>(0.669723)	0.0885064<br>(0.0812383)	-	-
Saccharomyces cerevisiae	2251/2018	40	40	40	40	39	2251/2018	0.0863600<br>(0.0821900)	7.77156e-16<br>(3.33067e-16)	0.000100452<br>(0.0000365022)	0.000216739<br>(0.0000839779)	0.0000140365<br>(0.00000627053)	0.000544323<br>(0.000195676)	0.0162625<br>(0.0131474)	0.000556758<br>(0.000379651)	0.000241010<br>(0.000173504)	0.307952<br>(0.303246)	0.0495351<br>(0.0469567)	0.168619<br>(0.164162)	0.301868<br>(0.300264)	0.0262503<br>(0.0244888)	0.553733<br>(0.552021)	-

Survival curves of inoculated larvae. Proportion of surviving larvae shown over time with 95% log confidence interval (lighter colors).

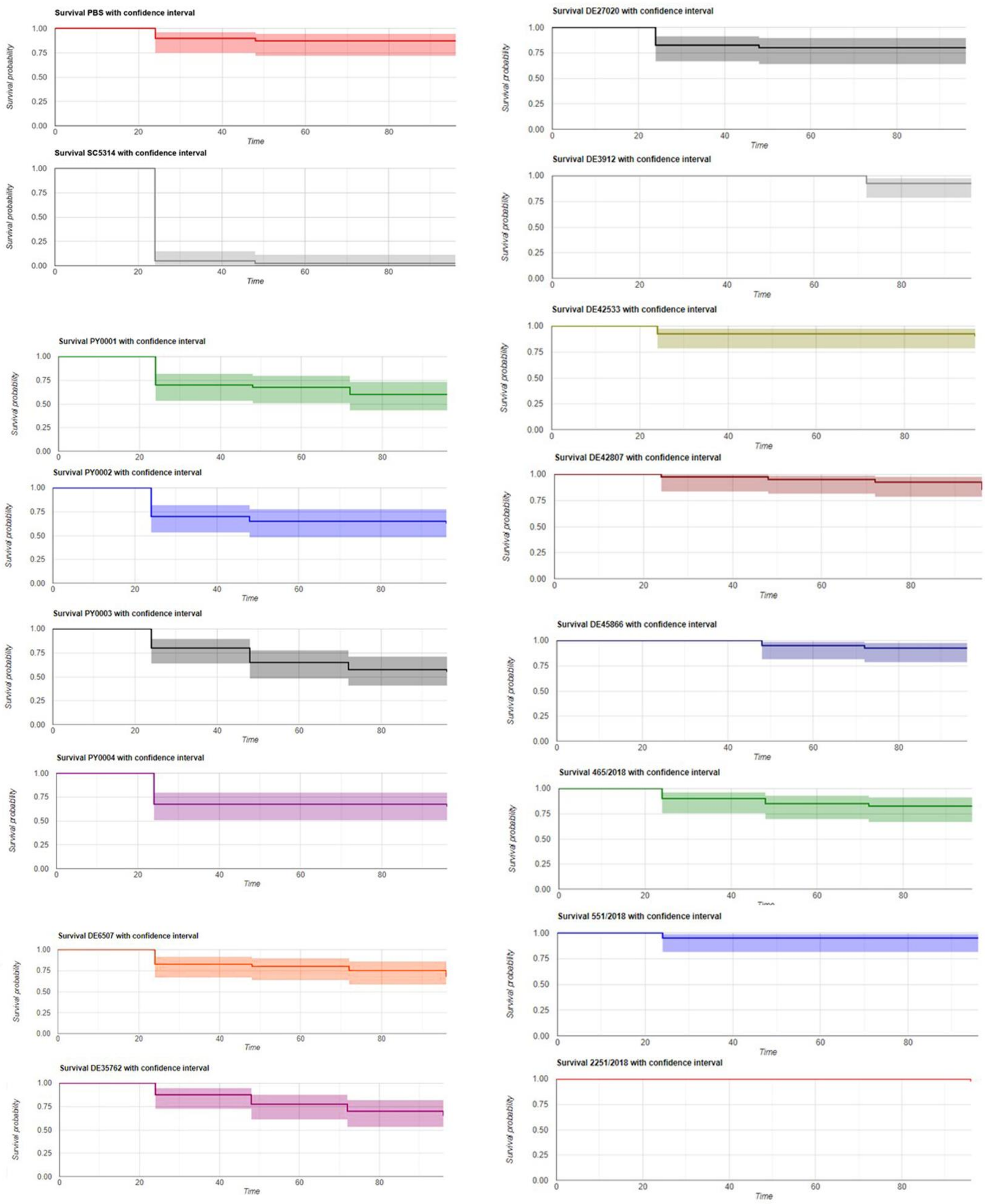

Supplement: Supplementary file 1 [file jof-07-00746-s001.zip › jof-1359670-SI.pdf]
